# A sugar flow model predicts cell dynamics, weight, and quality of tomato at varying sink–source ratios and temperatures

**DOI:** 10.1093/jxb/erag331

**Published:** 2026-07-04

**Authors:** Pieter H B De Visser, Jaap Molenaar, Paul C Struik

**Affiliations:** Wageningen UR, Greenhouse Horticulture, PO Box 644, Wageningen 6700 AP, The Netherlands; Wageningen UR, Biometris, PO Box 16, Wageningen 6700 AA, The Netherlands; Wageningen UR, Crop Systems Analysis, PO Box 430, Wageningen 6700 AK, The Netherlands; INRAE-Bordeaux, France

**Keywords:** Cell division, cell expansion, fruit growth, fruit load, fruit quality, metabolites, modelling, plant shading, sugar metabolism, temperature

## Abstract

A new tomato fruit model predicts cell numbers, cell sizes, sugar contents, and fresh weight. Transport of water and saccharides from the plant stem to fruit cells is computed following biophysical rules. The saccharide fruit sink is based on sugar metabolism, rates of cell division and expansion, and starch and cell wall dynamics. Osmotic and hydraulic potentials in cells and their vacuoles drive water import at a given cell wall extensibility. The interaction of demand and transport determines saccharide flow and biomass. We incorporated physiological responses to temperature, pruning, and plant shading. Existing and new parameters were calibrated with data from fruit heating and fruit pruning experiments of contrasting tomato cultivars. Model validation for different strategies of fruit heating and pruning, and plant shading was successful. Increased fruit temperature was shown to reduce fruit weight, as expected. Growth response to fruit pruning or shading was fully explained by changes in phloem sucrose concentration. Hydraulic conductivity of vascular tissue as well as sucrose and hexose carrier capacities were crucial fruit properties determining sugar flux. Model scenarios on knockdown of sucrose synthase and active hexose uptake affected sugar composition. The model creates an important step towards predicting fruit quality and taste under diverse growth conditions.

## Teaser

Temperature-driven sugar kinetics, cell division, and expansion were introduced in a merged tomato fruit model, which was able to account for sugar contents and fruit fresh weight of various cultivars.

## Introduction

Worldwide, tomato is the most important fleshy fruit produced in agriculture, with an estimated production volume of 192 Mt year^–1^ in 2023 (FAO, 2025). Tomato is also the most studied species for fleshy fruit development and quality, both as a model system and because of its economic value (Kimura and Sinha, 2008; Van der Knaap and Østergaard, 2018; Mauxion *et al*., 2021). A wide diversity of cultivars has been bred so far, reflecting a wide spectrum of properties, from fruit size to taste. Sugar dynamics have a major impact on growth, affecting the number and size of cells, texture, and sugar-related qualities such as sweetness, soluble sugars (Brix value), acidity, and mealiness. Completely mechanistic prediction of these traits requires a model on growth and sugar metabolomics at the cell scale taking into account water transport and fresh fruit weight increase. Obviously, the environmental factors light, CO_2_, temperature, and water supply are also essential to predict plant and fruit growth. Combining these external factors with internal, intrinsic factors in an eco-physiological model may account for the effects on carbon and water fluxes in fruits, with these .being the most relevant processes that explain fruit quality (e.g. Ho, 1996; Struik *et al*., 2005). Such a model is indispensable to answer research questions on quality issues that are relevant for commercial practice.

Many studies on tomato fruit show proper predictions of responses of fruit growth to varying temperatures and fruit loads using a descriptive model (Fanwoua *et al*., 2013), a regression or correlation model (Adams *et al*., 2001; Bertin, 2005), or a semi-mechanistic model (De Koning, 1994; Marcelis *et al*., 2009). Yet, these approaches did not incorporate the sugar supply pathways, the role of water transport, or the sugar kinetics in the fruit, and thus cannot predict the quality of fresh fruits and their Brix content in a mechanistic way.

Nowadays, more knowledge has become available on the computation of water and sugar flow into the tomato fruit. Chen *et al*. (2021) developed a fruit model that combined the simulation of biophysical processes (Liu *et al*., 2007) with a sugar metabolism model (Beauvoit *et al*., 2014) and was thereby able to predict sweetness and size of the fruit of several genotypes under various source supply regimes. Using their fruit model, Chen *et al*. (2021) surmised that the sugar carriers for sucrose and hexose and the magnitude of sucrose hydrolysis in the vacuole are promising candidates for breeding larger and sweeter tomato fruits. Interestingly, this has recently been put into practice by introducing specific invertase genes (Fernie and Martinez-Rivas, 2025). Although this example illustrates the strength of the model of Chen *et al.*, it misses a number of processes, which makes it less applicable in research and practice. First, there is no temperature dependency incorporated. Second, the model assumes one, big (pericarp) cell, ignoring cell division processes, while the developmental course of cell wall extensibility and membrane permeability—that results in sink strength for water and sugars—is described as a function of time and not related to the temperature-driven development rate. Other existing fruit models that do simulate cell division, cell endoreduplication, and cellular growth miss sugar metabolomics and temperature (Bertin *et al*., 2007; Baldazzi *et al*., 2019). Third, the model of Chen *et al.* does not explicitly quantify the proportion between the three used pathways of sugar import (diffusion, hydraulic conductivity, and active uptake), while Ho (1996) already suggested that at the end of growth, sugar is imported solely from the apoplast. McCurdy *et al*. (2010) found that suppressing the apoplastic carriers may decrease fruit weight by 14–30%. Fourth, the model of Chen *et al.* lacks an estimate of fat and protein production during glycolysis, an element that is required for wide applicability.

Since prediction of important fruit traits such as Brix content and dry matter content is possible with the model of Chen *et al*. (2021), that model will be the starting point of this study. We will add temperature as a crucial growth factor, and validate its effects using data from fruit temperature experiments. We will also add cellular dynamics since the division and potential expansion of cells affect the sink strength for carbohydrates, while sugars—mainly glucose—in turn influence the rate of cell division (Jin *et al*., 2009). Introducing the cells as sources of sink, the sinks for carbon and water align with the concept of sink strength for fruits as formulated by Ho (1996). The present cell model is simplified by merely simulating parenchyma cells, as is generally done for tomato fruit cell dynamics (Bertin, 2005). We will evaluate the most relevant growth processes for their dependency on temperature. The model is confronted with data of five tomato cultivars that were experimentally exposed to different temperatures and sugar supplies. In the Discussion, we reflect on the advantages and drawbacks of such a detailed model in the prediction of tomato fruit growth and sugar contents.

## Materials and methods

### Model concept and additions to existing models

Our modelling approach incorporates three different sets of processes: (i) biophysics and thermodynamics; (ii) enzyme-dependent sugar kinetics; and (iii) cell dynamics. Previous versions of model processes (i) and (ii) have been reported (Beauvoit *et al*., 2014; Chen *et al*., 2021). The aim of the integral model was to correspond to the virtual fruit concept, already described by Génard *et al*. (2007) and summarized in Fig. 1. All the model parts had to be merged to exchange their data at every time step (minute). The newly added cell dynamics module may provide new insights, since it uses the concept of sink strength, whereas the existing biophysical and kinetic models mainly describe the source, namely water and sugar supply, omitting a mechanistic simulation of the sink. The sink strength can be determined from the potential fruit growth resulting from unconstrained (i.e. not limited by resources) division and expansion of cells. Both growth processes are temperature dependent, and their development can be simplified to a logistic increment over thermal time. Thus, the one big growing cell as a sink in Liu *et al*. (2007) is replaced by the product of dividing cells and their expansion, described by logistic equations with thermal time as input. To avoid unnecessary model complexity, we only used the dominant cell type: the parenchyma cell in the pericarp. Despite such a simplification, we can address research questions on possible compensation of a lower cell number by a stronger cell expansion (e.g. Bertin, 2005; Okello *et al*., 2015). In short, the two main model changes were: (i) the introduction of temperature in the kinetic and developmental part of the model; and (ii) the use of cell number and cell size to estimate sink strength. Although a recent use of the model of Chen *et al.* within a plant model claimed that temperature influence had been added to predict effects of climate change, this only referred to the source supply and so temperature effects on fruit growth and sugar dynamics were discarded (Zhou *et al*., 2023).

**Fig. 1.**
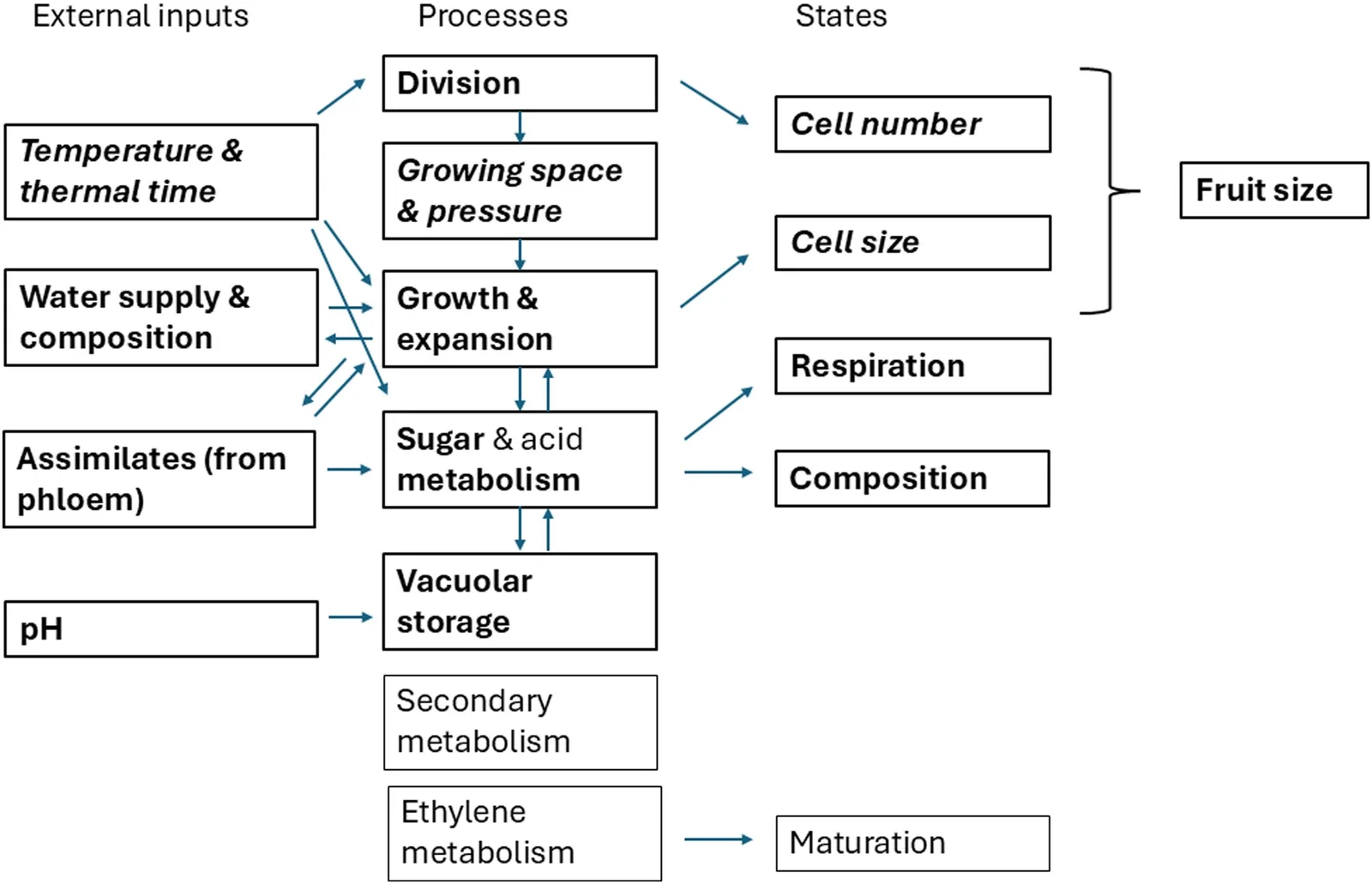
Relational diagram of processes in the model. The diagram is adapted, with permission, from Génard *et al*. (2007), indicating omitted parts (thin characters), existing parts (bold face), and newly added features (bold italics).

We implemented the following concrete model changes. (i) The kinetic rates of the used metabolic processes are now temperature dependent, by introducing the Arrhenius function, named ‘activity coefficient’, in each metabolic reaction rate equation. Its parameterization is derived from a Q_10_ value of 1.4, a generally accepted value for metabolism and respiration in horticultural crops (Van der Ploeg and Heuvelink, 2005). (ii) All developmental, time-related functions are now transformed into a dependency on thermal time: an increase in temperature will mean that cell division as well as cell expansion will stop sooner, and the solute reflection coefficient will decrease faster; the enzyme activities—described by Chen *et al.* as a function of time—will now decrease or increase faster. (iii) As for metabolic processes (Change i), both cell division and cell expansion show a higher activity at a higher temperature in the used temperature range, in addition to a higher rate of development with higher temperature (Change ii). (iv) New cells are new sinks for water and sugars because they add potential volume. (v) The sugar and water sinks of newly formed cells are equal to the number of new cells multiplied by the water and sugars required for their initial size and their potential expansion. (vi) The actual, momentary value of cell expansion is determined by the current availability of water and sugars. (vii) Potential cell expansion is model-wise expressed in terms of degree of cell wall extension. (viii) The loss of sucrose due to cell maintenance is accounted for. (ix) The activity of the hexose plasma membrane carrier decreases slowly and possibly linearly with fruit development, to allow more hexoses to arrive via the apoplastic sugar import. (x) The sugar flow to glycolysis results in biomass after subtracting respiratory and construction costs.

This approach enabled two new biophysical features in the model: (1) introduction of a mechanism whereby newly formed cells consume water and thus decrease the hydrostatic pressure in the fruit tissue, which facilitates an increase in water flow into the fruit; and (2) the potential cell size growth is a function of the degree to which the cell wall can stretch, making it a proxy for cell wall elasticity. With Feature 1, we add the role of cell numbers; with Feature 2, we can use observed cell sizes to parameterize the cell wall extensibility instead of using an empirical, time-dependent function from the literature. Given this potential sink, the actual cell growth depends on the availability of water and sugars in interaction with the strength of the sink, like a push and pull mechanism. All other mechanisms in the biophysical part of the model were similar to those in Liu *et al*. (2007).

Active uptake of hexose from the apoplast requires a transport carrier in the cell membrane. In the original model, its kinetics were delineated by a descriptive, negative exponential relationship with time. However, observations showed that hexose import becomes increasingly active in the course of fruit growth to compensate for the decreased symplastic uptake (Ruan and Patrick, 1995). In our model, alternative time courses for the carrier activity are suggested, and the involved curve parameters will be fitted to observation of fruit sugar contents. Note that if a hexose carrier is mentioned, we actually refer to the resultant of the activities of the putative carrier (as used by Chen *et al*., 2021) and of the apoplastic invertase, being triggered by the *Lin5* gene (Fridman *et al*., 2000).

### Modelled biophysical relationships

We used the biophysical tomato fruit model from Chen *et al*. (2021), this being the latest version as based on Liu *et al*. (2007), who had transformed the initial model for peach by Fishman and Génard (1998) into a tomato model. That version, in which Chen *et al.* and, previously, Beauvoit *et al*. (2014) also integrated a sugar kinetics model, is further referred to as ‘the original model’. The original enzyme reactions, differential equations, and variables used are provided in Supplementary Tables S1, S2, and S3, respectively. The original model simulates one big pericarp cell and divides the connected phloem tissue into apoplast and symplast. Our changes to the original model are explained below and shown in Fig. 2.

**Fig. 2.**
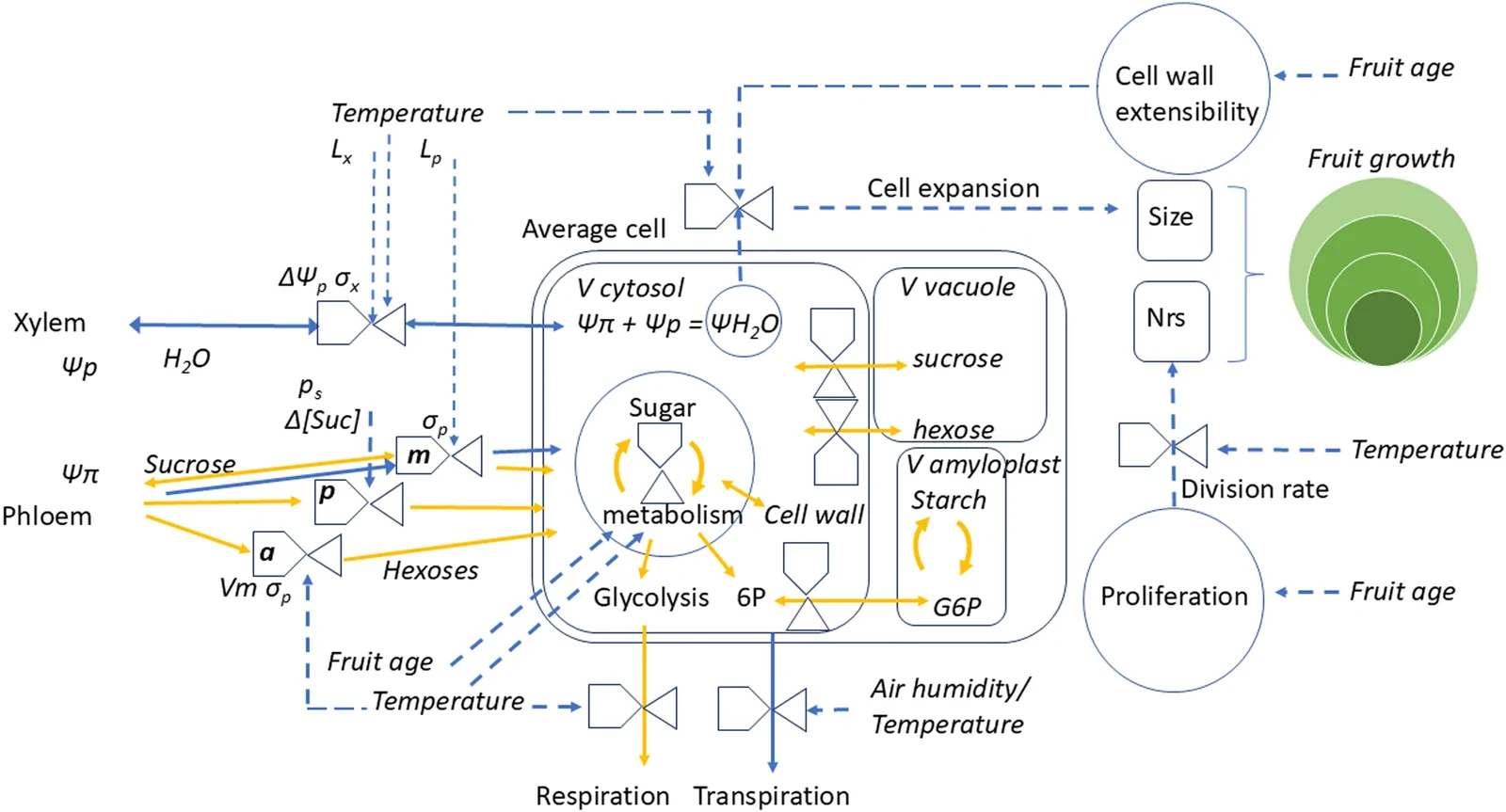
Forrester diagram of water and sugar flows relevant for modelled fruit growth processes. State variables that influence these flows are shown in italics. L, vascular hydraulic conductance; Ψ_p_, water potential; Ψ_π_, osmotic potential; Ψ_H2O_, hydrostatic potential; Δ(suc), sucrose difference between stem and fruit, σ, reflection coefficient of solute membrane; m, mass flow of water and sucrose; p, passive sucrose flow; a, active hexose flow; V_m_, capacity of active hexose transport; V, volume of cell compartment; subscripts p and x denote phloem and xylem, respectively.

In this section, we will refer to equations as listed in Supplementary Protocol S1, and the full model code is given in Supplementary Protocol S2 . According to the model of Fishman and Génard, the water supply from the phloem (Equation 1) to the fruit cells is thermodynamically driven by hydrostatic pressure differences between phloem and pericarp parenchyma cells, supposedly through the symplastic connections, namely the plasmodesmata. Sucrose that is transported by this water flow is defined as mass flow. When during fruit development the plasmodesmata gradually close—with full closure at 25 days after anthesis (DAA)—this symplastic transport from the phloem fully stops, and this gradual closure to the solutes is described by a membrane reflection coefficient increasing from 0 (fully open) to 1 (fully impermeable), as stated in the original model (Equation 7). The second form of water flow is from the xylem to the symplast, also governed by hydrostatic pressure differences, and which is ∼15% of the total water influx (see Equation 2).

The biophysical model computes the osmotic pressure from the concentrations of dissolved sugars, calculated per time step by the kinetic model. The water potential gradient is then used to calculate the bidirectional water flow between phloem and fruit symplast, by combining the equations of water supply (Equation 3) and potential, irreversible fruit cell expansion (Equation 4), rewritten to one solvable equation as done by Fishman and Génard (1998) (Equation 5). This fresh weight increase results in an amount of dissolved sugars, cell walls, starch, fats, and proteins corresponding to their calculated concentration in the previous time step, which leads to the actual sugar uptake and dry matter increment for the current time step. This uptake flux is used to update all concentrations within the sugar kinetics model, for use in the next time step.

The sugar is imported to the fruit in three ways: hydraulic conductivity, diffusion, and active uptake. The hydraulic conductivity of sucrose, further on referred to as mass flow, is computed by assuming a sucrose concentration equal to that in the supporting phloem sap (Equation 8). The diffusion is driven by concentration differences between phloem and symplast, and takes into account a conductivity of the supporting tissue (Equation 9). Apart from these two symplastic routes, dissolved sugar in the form of hexoses (glucose and fructose) is actively transported from the apoplast to the fruit symplast by means of hexose carriers in the plasma membrane (Equation 10).

The parameter values of the original biophysical model were taken and calibrated when necessary (see the Calibration section in the Results) and are provided in Supplementary Table S4A and B, respectively. The water and osmotic potential in phloem and xylem of the supporting vascular tissue were not known for our experiments, so their values were taken from studies on commercial cultivars with similar irrigation and substrate electrical conductivity conditions conducted by De Swaef and Steppe (2010). This also holds for the rate of fruit skin transpiration.

### Modelled sugar import

The parameter values for the import of sugars, realized by mass flow, passive diffusion, and active hexose uptake, were taken from the original model, and that version was based on sets of data summarized in Table 1. The maximum rate of active uptake of hexose is taken from the original model. However, since levels and the time course of simulated hexose concentrations did not match our observations, we adapted the active uptake function. Now the decrease of active hexose uptake with thermal time is assumed proportional to 1−σ_f_, where σ_f_ is the solute reflection coefficient between phloem vascular tissue and the fruit compartment, as is also the case for the computation of mass flow. This proportion is determined model-wise by fitting the predicted hexose concentrations to measured ones, varying the main calibration variables (see below).

**Table 1.** Overview of datasets and their use in model testing and basic parameterization

Cultivar	Data source	Modelled processes	Treatment factor/independent variable
Calibration			
Cappricia, Brioso	Okello *et al*. (2015)	Cell division and expansion rate, sugar contents	Temperature
Moneymaker Moneyberg	Chen *et al*. (2021) Fanwoua *et al*. (2012a)	Cell division and expansion rate, sugar contents	Sugar supply
Moneyberg	Experiment 1 in Fanwoua *et al*. (2012b)	Cell division and expansion rate	Temperature
Basic physiological data			
Moneymaker	Experiment 1 in Biais *et al*. (2014), used for calibration in Beauvoit *et al*. (2014) and Chen *et al*. (2021)	*V*_max_ and *K*_m_ of enzymes and carriers of hexoses and sucrose, rates for construction and degradation of starch and cell wall	Sugar supply
Moneymaker, Dirk	Liu *et al*. (2007) De Swaef and Steppe (2010)	Hydraulic conductance and size of transport tissue, arriving at osmotic pressure and turgor in fruit	Concentration of sucrose and other solutes in phloem
Moneymaker	Chen *et al*. (2021)	Hydraulic conductance, cell wall extensibility, membrane solute reflection	Time
Moneymaker	Colombié *et al*. (2015)	Carbon allocation to fats and proteins	Time, sugar flux to glycolysis

### Cell division and expansion in the model

The cell division model generates new cells that add volume and thus release of turgor pressure in the existing volume (Fig. 1). Subsequently, existing and new cells grow irreversibly in volume according to the Lockhart equation (Equation 4 in Protocol S1; equation 13 in Fishman and Génard, 1998), using turgor, water availability from the phloem and xylem, and cell wall extensibility, and the demanded water volume is diminished with the volume of water transpiration and volume (V_n_) of new cells.

The rates of cell division and expansion are described by Richards curves with thermal time (Equation 19), which were multiplied by an activity coefficient (Equation 16) with a Q_10_ of 1.4 as first estimate for higher activity at higher temperatures (Van der Ploeg and Heuvelink, 2005). For expansion, the Richards parameter values are assumed to reflect a potential, irreversible volume increment. This approach thus replaces the product of extensibility of the cell wall (Ф) and the fruit hydrostatic pressure (P_f_) minus the threshold value for irreversible expansion (Y) in the Lockhart equation 13 in Fishman and Génard (1998), allowing a biologically realistic cell expansion growth pattern that reflects the degree of extensibility during cell development. The advantage of this approach is that this extensibility, which is very hard to measure directly, can be indirectly estimated by tuning the Richards parameters to observed actual expansion. For this potential extensibility, the data of an experiment with two fruits per truss (i.e. low fruit load) were used, representing fruit growth that is potential, being sink limited and having unconstrained availability of water and sugars. The resulting value for Ф can be evaluated by comparison with reported values in the literature.

### Modelled sugar metabolism

The incoming sucrose flows are input into the original, kinetic model for sugar metabolism in the tomato pericarp tissue. We adopted the same time courses of the enzyme activities as were measured by Beauvoit *et al*. (2014). Similarly, we assumed that growth of the sugar sinks in the kinetic model (cell walls, starch pool in amyloplast, and dissolved saccharides in the vacuole) is proportional to the fresh weight growth computed with the biophysical model.

The concentrations of cell wall material and starch—in hexose equivalents per gram fresh weight—are calculated using the construction and degradation rates as fitted in the original model to the observations reported in Biais *et al*. (2014). Similarly, the original ordinary differential equations (ODEs) of the kinetic model were adopted to simulate sugar metabolism of the original model. For the evaluation of the ODEs, we applied the 23S ODE solver of MATLAB, version 2023. All kinetic reactions are described as Hill (or Michaelis–Menten) equations using the *V*_max_ and *K*_m_ values for the specific reaction.

After the cascade of sugar transformations and resulting concentration changes in the cytoplasm, vacuole, and amyloplast, the remainder of the sugars passes to glycolysis in the form of fructose-1,6-biphosphate. The original kinetic model did not specify the fate of this fructose-1,6-biphosphate. Our model uses this carbon for the respiratory costs of maintenance and biomass construction, and stores the remaining carbon in a dry matter pool—consisting of fats and proteins—and an organic acid pool that are built downstream of the sugar kinetics (Fig. 2). The respiratory costs of the biomass (the pools of fats, proteins, cell walls, and starch) comprised a fixed 30% of the carbon allocated to their growth and a daily maintenance respiration of 2% of the carbon in this biomass at 21 °C (both estimates are based on the carbon construction costs used in Heuvelink, 1995). The resulting amounts of dry matter were of the same order of magnitude as reported by Colombié *et al*. (2015).

For all simulations, the day and night phloem sucrose concentrations from Chen *et al*. (2021) (0.054 g g^–1^ and 0.126 g g^–1^) are applied as constant 12 h values, as are the values of hydrostatic pressure in the xylem, derived from a tomato trial with optimum water supply (−0.65 MPa and –0.05 MPa; see De Swaef and Steppe, 2010), whereas day and night air temperatures are at ±1 °C from the imposed daily temperature setpoint of the selected treatment.

### Calibration

The datasets and the associated fitted or validated model processes are listed in Table 1. We examined four tomato (*Solanum lycopersicum* L.) cultivars: Moneymaker, Moneyberg, Cappricia, and Brioso.

The equations and parameter values of the combined biophysical and sugar kinetics fruit model, fitted for the cultivar Moneymaker by Chen *et al*. (2021), were tested in the modelling environment in MATLAB—being copied from the Copasi version in Beauvoit *et al*. (2014) and updated by values in Chen *et al*. (2021) for the Michaelis constant *K*_m_ (for the tonoplast carriers for sucrose, glucose, and fructose, and for acid invertase and neutral invertase), the Michaelis constant *K*_i_ for neutral invertase of glucose and fructose, and the ratio of forward to reverse activity for phosphoglucomutase (rVfVfPGM) and for UGPase (rVfVfUGPase). Remarkably, some parameters required recalibration to fit the simulated time course of fresh fruit weight and sugar concentrations to the observed values (Chen *et al*., 2021). The fitted parameters were phloem hydraulic conductivity (*L*_p_), the maximum mass flow rate, and the maximum value and time course of active sugar uptake. In addition, the vapour permeation coefficient of the fruit skin (ρ) was increased to match fruit transpiration estimates by Liu *et al*. (2007) that ranged from 0.1 g d^–1^ (10 DAA) to 0.6 g d^–1^ per fruit (55 DAA), totalling 17.5 g over the growth period.

As for tomato cultivars other than Moneymaker, the parameter values in the biophysical model that were assumed to be cultivar-specific were (i) *L*_p_, (ii) the initial, maximum value for active sugar uptake (c1 in Equation 10), (iii) the Q_10development_ for increase of phloem solute reflection σ_p_ with time (see Equation 7), (iv) the activity coefficient of cell expansion for temperature (Equation 17), and (v) the maximum and shape parameter of the Richards equation for cell expansion (Equation 19). The Richards parameter values for cell division (Equation 19) were set to reach observed cell numbers prior to calibration. A cultivar-dependent set of 3–5 parameters was calibrated per cultivar, using a genetic algorithm, to reach fruit weight observed at six time points. All other parameters were afterwards manually fitted in order to reach observed concentrations of sucrose, fructose, and glucose in the pericarp tissue, except for the temperature treatment of cv. Moneyberg, where sugars were not determined.

### Data on temperature response

The temperature sum to reach fruit ripening and the base temperature were taken from the studies on the temperature experiments on the three cultivars as detailed below. As mentioned in the model concept section, the time course of all developmental processes, such as the solute reflection coefficient (σ), the *V*_max_ of all metabolic reactions, and the cell wall and starch synthesis rates were converted to thermal time. The developmental functions are described in Supplementary Tables S5 and S6, and in Supplementary Protocol S1. For cvs Moneymaker and Moneyberg [datasets in Chen *et al*. (2021) and Fanwoua *et al*. (2012a) were solely on source supply treatments], the parameter values on temperature dependencies were assumed equal to the fitted ones for the Moneyberg heating experiment. The new model on cell dynamics was calibrated using a genetic algorithm (GA; see the section on statistics) fed with the data on cell number, cell size, and fruit weight as observed (Table 2). For this, the parameters in the temperature-dependent Richards equations for cell division and expansion (Equation 19) were fitted to follow the growth curves of cell numbers and cell expansion, and the values for maximum cell division and expansion rate were fitted by adapting the Arrhenius coefficient (Equation 16). The data for the temperature dependencies were derived from the following experiments. (i) For tomato cv. Moneyberg, in a temperature experiment (UNIFARM greenhouse facility in Wageningen, The Netherlands) conducted in 2010, all trusses were enclosed in transparent Perspex cuvettes (length 40 cm, diameter 13 cm) where the temperature of the actively forced flow of incoming air was either the default greenhouse air temperature of on average 21 °C or air heated to +4 °C using a sensor-controlled heating device. The plants of the different treatments were always situated in the same greenhouse compartment, so the plants experienced the same default air temperature. Observations consisted of time courses of fruit fresh and dry weight, as well as the volume and weight of pericarp tissue, and the number and size of pericarp cells (Fanwoua *et al*., 2012b). (ii) For cv. Cappricia (Rijk Zaan B.V., De Lier, The Netherlands), the experimental design was similar to that for cv. Moneyberg. The experiment was conducted at the UNIFARM greenhouse facility in Wageningen, The Netherlands, in 2011. Similarly, observations consisted of the time courses of fruit fresh and dry weight, as well as volume and weight of pericarp tissue, and number and size of pericarp cells. In addition, sugar contents of pericarp tissue were determined for six moments during growth of the truss (Okello *et al*., 2015). (iii) For cv. Brioso (Rijk Zwaan B.V., De Lier, The Netherlands), a similar experiment was synchronously performed as for cv. Cappricia, and similar observations were done (Okello *et al*., 2015).

**Table 2.** Observed and modelled fruit weight, fruit cell number, cell size, and hexose concentration

	Validated treatment		Calibrated treatment	
	High sugar supply (low fruit load)		Mild sugar supply (default fruit load)	
cv. Moneyberg	Observed	Modelled	Observed	modelled
Fruit fresh weight (g)	98 (1.42× default)	97	69	69
Fruit cell number (×10^6^)	4.67 (1.19× default)	4.66	3.93	3.93
Fruit cell weight (g×10^–6^)	21.0 (1.19× default)	20.8	17.6	17.6
Hexose content (µmol g^−1^ FW)	118	139	118	123

	Low sugar supply (60% less PAR*^a^*)		Default sugar supply	
cv. Moneymaker	Observed	Modelled	Observed	Modelled
Fruit fresh weight (g)	42 (0.64× default)	43	66	67
Fruit cell number (^×^10^6^)	n.a.^a^	6.06	n.a.	7.25
Fruit cell weight (g×10^–6^)	n.a.	7.1	n.a.	9.2
Hexose (µmol g^−1^ FW)	100 (0.83× default)	108	120	123

	Calibrated treatment		Calibrated treatment	
	Temperature +4 °C		Default temperature	
cv. Moneyberg	Observed	Modelled	Observed	Modelled
Fruit fresh weight (g)	62 (0.64× default)	62	97	97
Fruit cell number (×10^6^)	5.7 (0.79× default)	6.2	7.2	7.3
Fruit cell weight (g×10^–6^)	10.9 (0.81× default)	10.0	13.5	13.3
Hexoses (µmol g^−1^ FW)	n.a.	97	n.a.	110

cv. Cappricia	Observed	Modelled	Observed	Modelled
Fruit fresh weight (g)	66 (0.88×default)	66	75	75
Fruit cell number (×10^6^)	22.5 (1.01× default)	22.2	22.2	22.4
Fruit cell weight (g×10^–6^)	2.9 (0.85× default)	3.0	3.4	3.3
Hexoses (µmol g^−1^ FW)	130	144	129	138

cv. Brioso	Observed	Modelled	Observed	Modelled
Fruit fresh weight (g)	28 (0.93× default)	27	30	30
Fruit cell number (×10^6^)	13.6 (1.15× default)	13.4	11.8	12.0
Fruit cell weight (g×10^–6^)	2.1 (0.84× default)	2.0	2.5	2.5
Hexoses (µmol g^−1^ FW)	140	156	130	129

The difference from the experimental reference treatment is indicated in parentheses. Data and results of model calibration (calibrated per cultivar) are shown with grey shading.

^*a*^PAR, photosynthetically active radiation; n.a., not available.

### Data on sugar supply response for calibration and validation

The data on sugar supply response were derived from two reported experiments imposing a high and a low sugar supply, respectively. Dataset 1 was derived from an optimal light and a shading treatment of cv. Moneymaker, where incoming photosynthetically active radiation was reduced by 60% by shading individual plants (Biais *et al*., 2014). This treatment was shown to result in a 25% reduction of the sucrose concentration in the supporting phloem (Chen *et al*., 2021). Time courses of fruit dry and fresh weights as well as concentrations of sucrose, glucose, fructose, cell wall materials, and starch in the pericarp tissue were available, but those for fruit cell numbers and sizes were missing. Dataset 2 for validation of the response of the model to sugar supply was derived from a default and a low fruit load treatment, enabled by keeping five or two fruits, respectively, on each truss of cv. Moneyberg (Fanwoua *et al*., 2012a). For this dataset, time courses of fruit fresh and dry weights were present, but the number and size of pericarp cells as well as sugar contents were only determined at harvest. In dataset 2, no data on phloem sucrose concentrations were present.

### Fitting procedure for calibration and validation

The differences between observed and simulated time courses of fresh weight development were reduced by optimization with respect to a selection of parameters in the fruit model. The optimization was conducted by a GA in MATLAB, simultaneously for control and temperature treatment, and per tomato cultivar to calibrate temperature-related parameters as well as general parameters. The GA per cultivar was ended when the last 10 iterations showed no further improvement in the goodness of fit. The selected parameters to be varied in the optimization were phloem hydraulic conductivity *L*_p_, the temperature coefficients for cell expansion and solute reflection development, two Richards parameters that determine the time course of cell expansion, the maximum mass flow rate, and a decay parameter for active sugar uptake. For the sugar kinetics model, all parameter values were taken from the original model. The differences between observed and simulated fresh weights and hexose concentrations were expressed by the mean absolute error (MAE) and the relative root mean square error (RRMSE).

A different procedure was used to fit the model when it comes to its predictions for varying sugar supply. For cv. Moneymaker, the calibration with the GA was used to fit the model to the temporal pattern of fruit growth and sugar contents in the default treatment. Subsequently, their shading treatment was used for validation. For this, the reported decrease in phloem sucrose was input to the model. Also for the cv. Moneyberg, the model was fitted for the default treatment (Fanwoua *et al*., 2012a). For this, all the parameter values of the cv. Moneyberg temperature simulation were used with two exceptions, given the different cultivation and growth year (see Fanwoua *et al*., 2012b): hydraulic conductivity of the phloem vascular tissue and cell division rate were manually tuned to reach a fruit weight, cell number, and hexose content as observed (only the values at breaker stage were present). Similar to cv. Moneymaker, the sugar supply treatment—here the low fruit load—was used for validation. In contrast to cv. Moneymaker, for cv. Moneyberg the phloem sucrose concentrations were not known: for the default we used the values reported by Chen *et al*. (2021) and for the low fruit load we searched its value iteratively in order to reach simulated fruit weight corresponding to observed fruit weight.

### Model sensitivity to sugar uptake approach

The sensitivity of model output to the approach on how sucrose and hexoses are entering the fruit was tested. The following approaches were conducted. (i) Computation of active hexose uptake according to the *V*_maxup_ with exponential decay as used and parameterized by Chen *et al*. (2021), instead of our function using 1–σ_f_ as a proxy for the decline. (ii) The decrease of the active, apoplastic hexose uptake with thermal time is assumed linear, with some activity left at the end of fruit growth. The slope of this curve is tested for its effect on the time course of hexose concentrations. (iii) An upper threshold for sucrose mass flow is incorporated so the computed sucrose influx never exceeds 25 nmol min^−1^ g^−1^ FW as reported by Beauvoit *et al*. (2014).

The suggested scenarios were studied for their impact on sugar content and fruit size.

### Modelled scenarios

The following scenarios were simulated with the model calibrated for cv. Cappricia. (i) Increasing both temperature and source supply to test unconstrained growth at 25 °C. At a crop temperature of 21 °C (used in the referenced experiments, generating the default phloem sucrose concentration), an increase in fruit temperature will accelerate fruit development and increase sink strength per time interval. We assumed that the increased sugar supply resulting from a lower fruit load could better address the increased sink demand and thus lead to fewer small fruits than normal at increased temperatures. (ii) Reducing the rate of *V*_maxup_ (the activity of the hexose carrier from apoplast to fruit cells) by 50% or 100%. This was already done *in vivo* by McCurdy *et al*. (2010), but consequences for fruit size and sugar content could also be demonstrated model-wise. The hexoses involved were formed by cell wall invertase from phloem-derived sucrose, and reduced import may considerably decrease fruit growth. When the fruit is fully switched from symplastic to apoplastic sugar input, fruit growth fully relies on these hexoses. (iii) Silencing the enzyme sucrose synthase (Susy) is claimed to promote sweetness (i.e. fructose content) of the fruit (Fernie and Martinez-Rivas, 2025). Since our model possesses the necessary pathways, we will predict the effect of a 100% silenced Susy enzyme on the dry matter, hexose, and fructose content of the fruit.

As for model sensitivity, the impact on cell dynamics, sugar content, and fruit weight and size were studied.

## Results

### General

Observed fruit weights were different between cultivars in the datasets used for calibration (Table 2). Cv. Moneyberg is a large-fruit cultivar (fruit weight >100 g) that, however, performed poorly in the experiment. Cv. Cappricia is a medium-sized cultivar and cv. Brioso a cocktail-tomato cultivar. Their fruit weights were in line with breeders’ specifications. The manipulation of source supply resulted in fruit weight responses of the same order of magnitude for the two datasets used, with cv. Moneyberg increasing by 42% at lower fruit load and cv. Moneymaker decreasing by 36% because of shading (Table 2). Interestingly, the rise in temperature resulted in equal relative reductions of fresh weight between cultivars but different responses for the number of cells (Table 2). This obviously led to different parameter values in the calibration for the different cultivars, and will be detailed below.

### Calibration of water and sugar flow

Before calibration, existing parameter values and/or model mechanisms had to be changed to ensure that the model generates realistic values for sugar, cell wall, and starch contents, osmotic potential, and fruit weight. Firstly, we aimed at correct simulation of osmotic potentials in phloem and fruit, as found by Liu *et al*. (2007) and De Swaef and Steppe (2010) by adjusting the contribution of other ions in the fruit to −0.5 MPa (instead of −0.35 in Chen *et al*., 2021 for their optimal growth conditions, applying −0.5 MPa for drought only) and changing the vapour permeation of the fruit skin well above the value of 0.162 g cm^−2^ h^−1^ MPa^−1^, reported by De Swaef and Steppe (2010) in order to realize transpiration levels as simulated by Liu *et al*. (2007) (see Supplementary Table S4B). This promoted mass flow and gave realistic values for turgor, osmotic and xylem water potential (Fig. 3). Still, hexose concentrations and fruit growth increased in the first 20 d and subsequently decreased too much, so we changed the existing dramatic decrease of both mass flow and active uptake. To depress the peak concentrations, we maximized the sucrose transport by mass flow to a threshold of 25 nmol sucrose min^−1^ g^−1^ FW as given by Beauvoit *et al*. (2014) for their sucrose influx. Additionally, the active uptake was modelled as a decreasing, logistic pattern over time, similar to the decrease of mass flow, being one minus the solute reflection coefficient σ_f_ (see Supplementary Table S4B for details). For cv. Moneymaker, a linear decrease of mass flow and active flow was used to fit the almost constant hexose concentrations in the fruit (Supplementary Table S4B). For all cultivars, the simulated starch contents with time were too low, so the starch synthesis coefficient *k*_SS_ was increased to fit the observations of Beauvoit *et al*. (2014). Finally, the phloem hydraulic conductivity (*L*_p_) was significantly raised to reach realistic fruit fresh weights with time. *L*_p_ was found to be one of the most important parameters for fitting the model for different cultivars (Chen *et al*., 2021). So, its value was subsequently subject to our further calibration.

**Fig. 3.**
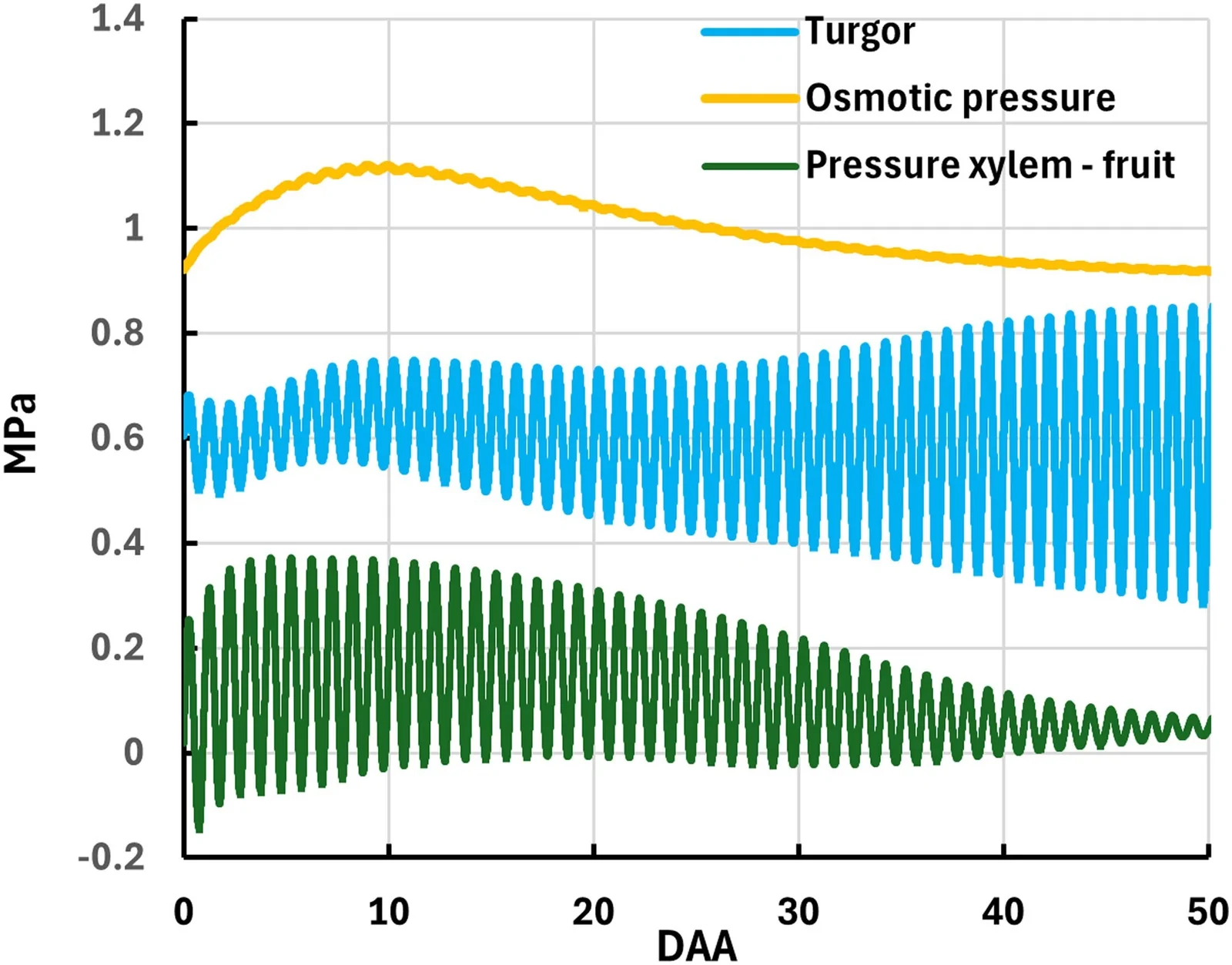
Modelled values of turgor, fruit osmotic pressure, and water pressure difference between xylem and fruit (all in MPa). Simulations were done for the control treatment of cv. Cappricia.

The calibrated, cultivar-specific parameters were *L*_p_, the parameters and activity coefficient of the temperature-dependent logistic cell division and expansion rate (see next paragraph), the upper threshold for mass flow of sucrose (explained above), the temperature coefficient for the increase of the solute reflection in phloem vessels (σ_f_), affecting sugar influx, and, finally, the initial maximum and the decay rate of active uptake (all adapted parameters are listed in Supplementary Table S4B). The general parameters (i.e. those that were not cultivar specific) that were calibrated—weighting the data of all cultivars equally—were the vapour permeation coefficient of the fruit skin ρ, the osmolarity of other ions π_F0_, the starch synthesis rate, the upper threshold of mass flow, the decay rate of active hexose uptake, and the four Richards parameters on cell dynamics (see also Supplementary Table S4B).

### Calibration of cell division and expansion for the default and heating treatment, and resulting sugar content

For cv. Moneyberg, application of the Richards equation for the number of cell divisions led to an early appearance of the maximum division rate at the higher temperature, and a concomitant decrease of the total number of cells. This early rate and low final value are inherent properties of the equation used, and the newly added ‘activity coefficient’ approached the value of 1 according to the GA fit. For the other cultivars, the activity coefficient had to be increased considerably to give similar (cv. Cappricia) or higher (cv. Brioso) simulated cell numbers in the heating treatment compared with the default treatment (see values for cell division in Table 3). Values of the calibrated Richards parameters and the temperature (activity) coefficients are listed in Supplementary Table S4B.

**Table 3.** Estimated value of the activity coefficient (equation 16 in Supplementary Protocol S1) at 25 °C relative to 21 °C organ temperature

Temperature response 25 °C/21 °C	Cappricia	Brioso	Moneyberg
TT solute reflection*^a^*	1.14*^b^*	1.06*^b^*	1.25*^b^*
Cell division	1.21*^c^*	1.43*^c^*	1.00*^c^*
Cell expansion	1.74*^b^*	1.87*^b^*	1.00*^b^*
Base temperature (°C)	3	3	5.7

The *V*_max_ of enzyme reactions, its change with thermal time, as well as maintenance were not fitted but are based on a Q_10_ of 1.4 (Van der Ploeg and Heuvelink, 2005), giving an activity coefficient of 1.14.

^*a*^TT (thermal time) of membrane solute reflection.

^*b*^Calibrated with GA

^*c*^Calibrated to fit observed cell numbers.

For cv. Moneyberg, the cell expansion responded similarly to its cell division by speeding up the expansion rate, yet realizing smaller cell size at higher temperatures, and the activity coefficient for potential cell size growth had no effect because expansion was supply limited. The cell size of the other cultivars was similarly lower (−16%) due to heating; yet, this resulted in slightly higher activity coefficients (Table 3). This shows that, according to the biophysical model, the supply of water and sugars may have been reduced at the higher temperature, and that for these two cultivars a higher degree of extensibility (the potential expansion is the driving sink and pulling force for water and, thus, sugars transported in the water by mass flow) was enough to arrive at the observed size.

Fresh weight growth of the fruit was calculated from the simulated combination of fresh weight growth of the average fruit cell and their numbers per fruit (see Fig. 4 for the rates of cell division and expansion for cv. Moneyberg). For agreement with the observed temporal pattern of fruit weight increment, the shape parameters in the Richard equations on cell expansion were slightly changed, following the GA fitting.

**Fig. 4.**
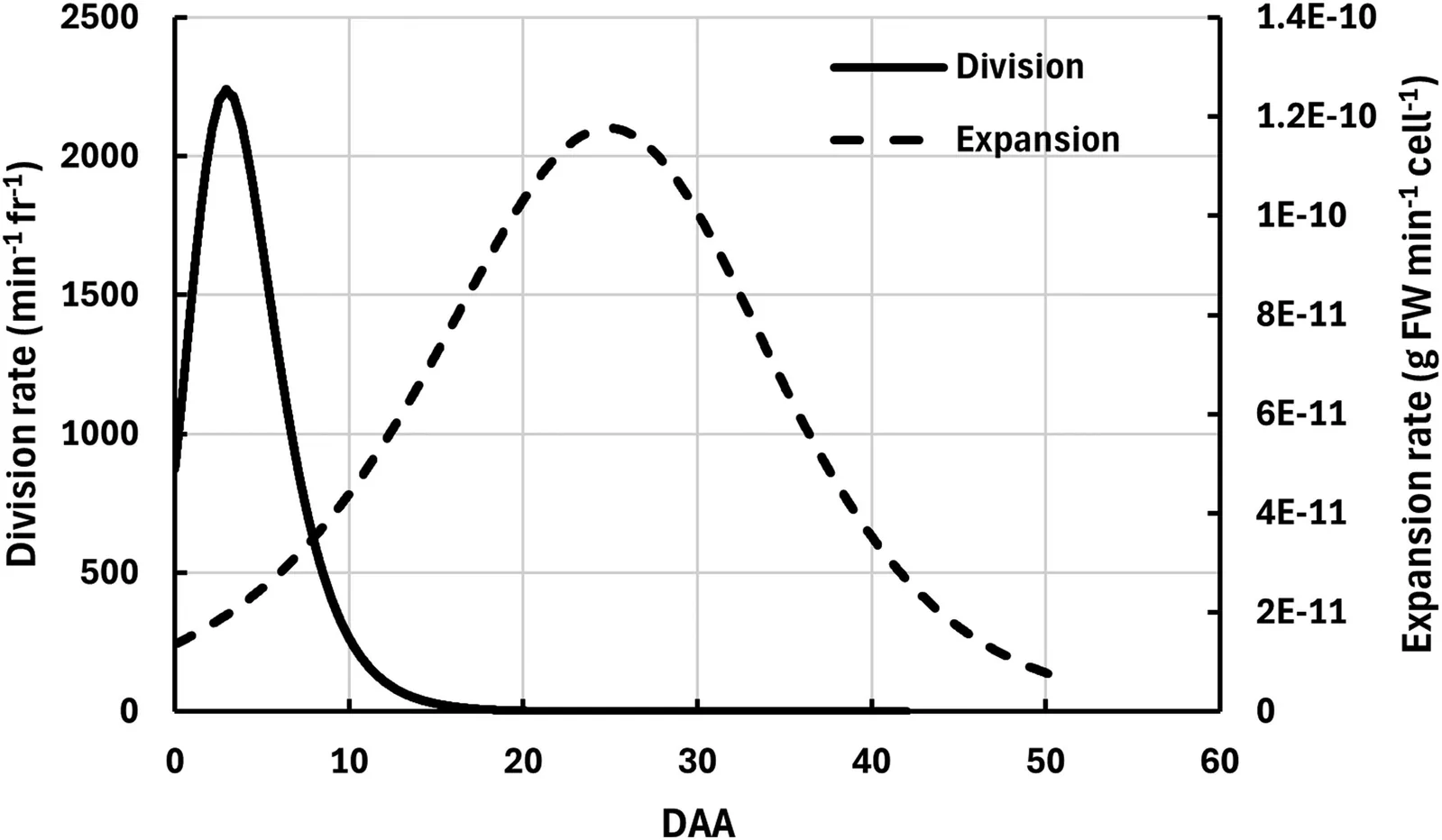
Simulated cell division and expansion rate per minute, at 21 °C for cv. Cappricia.

The resulting actual cell expansion and its multiplication by cell numbers led to a fruit fresh weight that agreed with the observations for all three cultivars (Fig. 5). The values for MAE and RRMSE were fairly low (Table 4), due to a good fit and because we used smoothed curves on observed fresh weight growth instead of measured, variable values per time step. The predicted hexose content agreed fairly well with the observations (Table 4; Fig. 6).

**Fig. 5.**
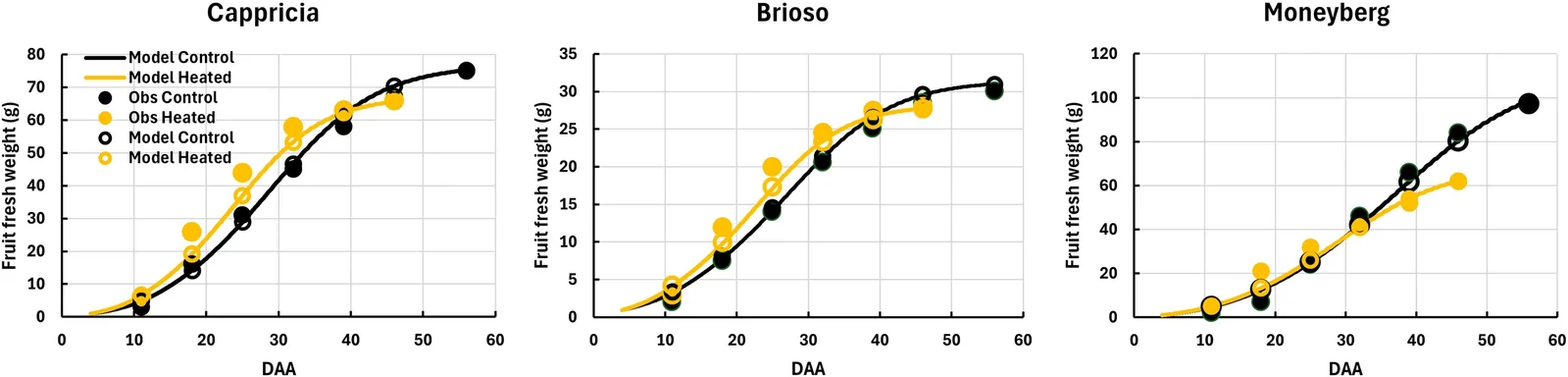
Simulated development of tomato fresh weight (g). Simulations were carried out for average air temperatures of 21 °C (Control) and 25 °C (Heated) for the cultivars Cappricia, Brioso, and Moneyberg. Simulations: curves and open circles, observations: filled circles.

**Fig. 6.**
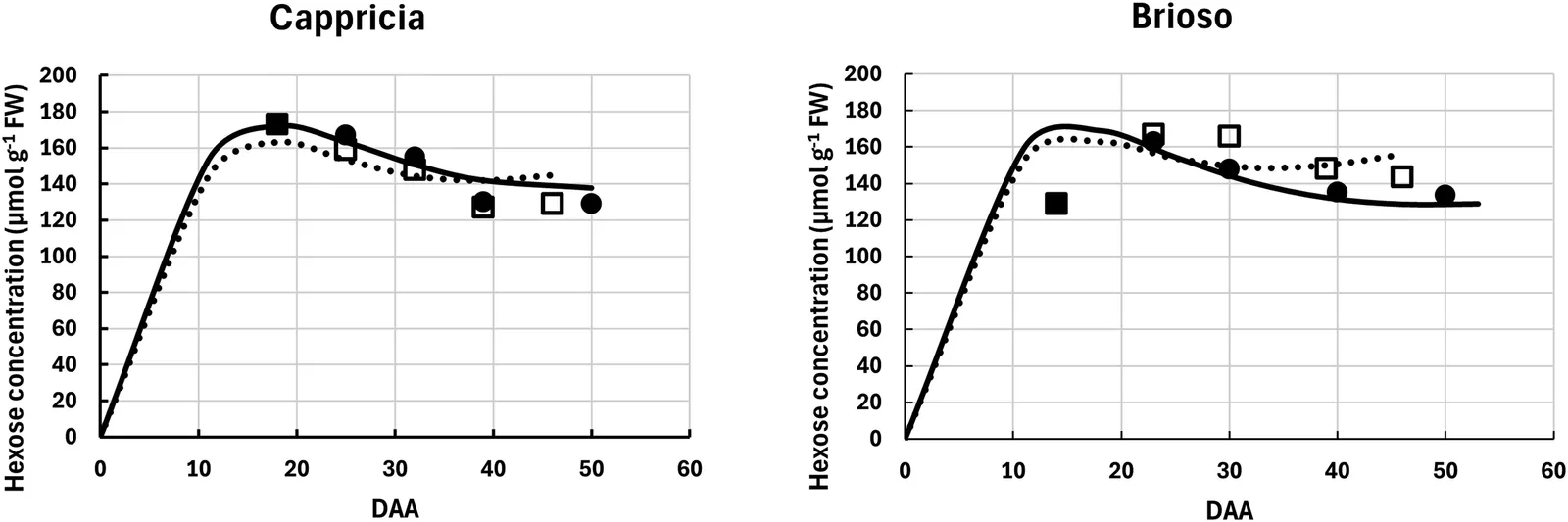
Hexose concentrations (in µmol g^−1^ FW) in the fruits of the treatments where time courses of hexose were examined. Observations: filled circles, control; open squares, heating treatment; open circles, shading treatment. Model results: continuous line, control; dotted line, heating treatment; dashed line, shading treatment. NB: observed values of control and treatment often overlap.

**Table 4.** Mean absolute error (MAE) and relative root mean square error (RRMSE) between the observed and simulated fruit fresh weight (FFW in g) and hexose concentration (HEX in µmol g^−1^ FW) of the studied cultivar×environment combinations

Cultivar and treatment	MAE	RRMSE	MAE	RRMSE
FFW	Control		Heated	
Cappricia	−0.88	0.05	3.23	0.10
Brioso	−0.92	0.06	1.09	0.09
Moneyberg	−0.85	0.08	1.54	0.09
HEX				
Cappricia	−6.19	0.08	−4.17	0.08
Brioso	−3.20	0.10	−3.74	0.13
FFW	Control		Source supply	
Moneymaker	0.22	0.09	−0.47 (shaded)	0.10
Moneyberg	−0.07	0.00	−0.93 (fewer fruits*^a^*)	0.01
HEX				
Moneymaker	1.54	0.05	4.69	0.06

^*a*^In the low fruit load treatment, the results for FFW were based on the final harvest only.

### Validation of modelled sink–source effects

For the fruit load experiment of cv. Moneyberg, the phloem hydraulic conductivity was the single parameter value to calibrate relative to the parameter set of the heating experiment—performed in another year and greenhouse—to model the fresh fruit weight time course as was observed in the control (default) treatment. Subsequent prediction of the low fruit load treatment was done by stepwise increasing the phloem sucrose concentration in the model. At a concentration increase of 19%, the modelled final fruit weight matched the observation precisely (Table 2; Fig. 7A). Typically, this 19% was also observed for the cell size and cell number increase following fruit pruning. The modelled fresh weight and hexose content were not sensitive to a forced increment of cell number to the observed level of 119% relative to the control. Hexose content slightly increased because of the higher phloem sucrose resulting from lower fruit load; yet, such an increase was not shown in the observations (Table 2). Surprisingly, in the same study, Fanwoua *et al*. (2012a) observed such an increase for another cultivar but not for cv. Moneyberg. Moreover, their fruit load treatments did not significantly affect the studied starch, glucose, and fructose content, or the sucrose–hexose ratio.

**Fig. 7.**
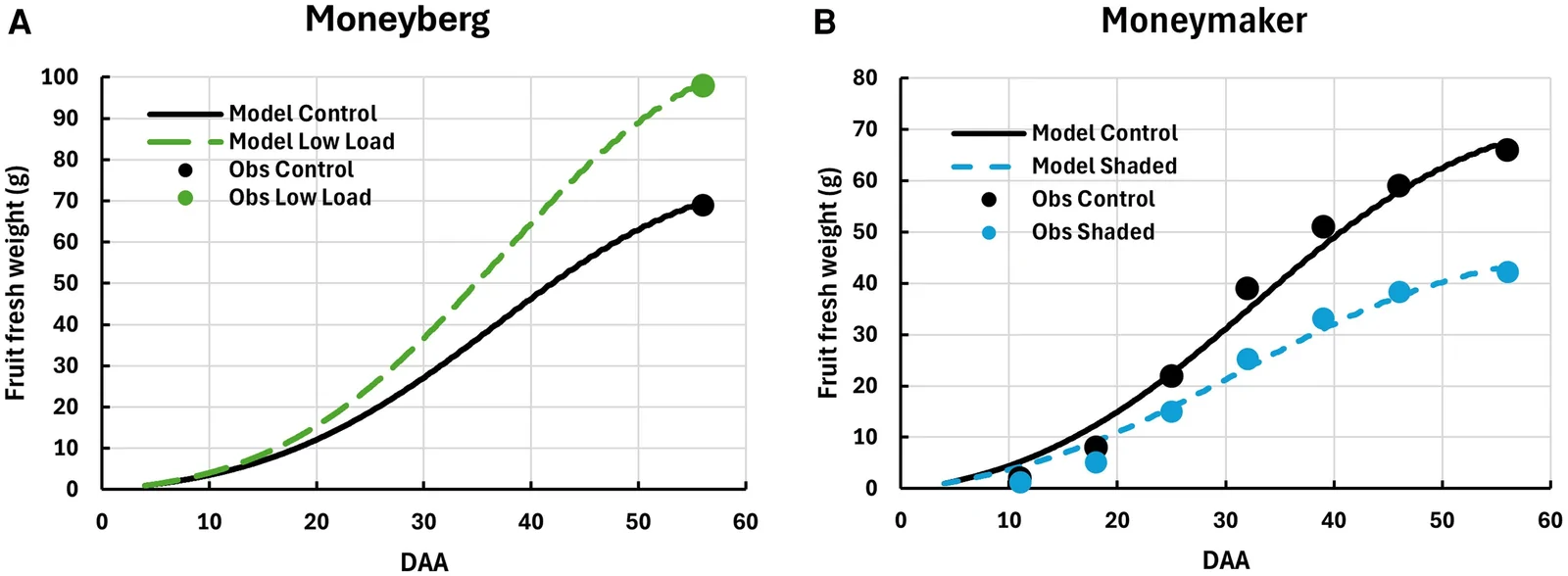
Modelled and observed fruit fresh weight (g). (A) cv. Moneyberg with default (control with five fruits per truss) or low fruit load (two fruits per truss). (B) cv. Moneymaker in control and shading treatment. Continuous lines are the values from the calibration treatment, and coloured, dashed lines from the predicted treatment.

For cv. Moneymaker, the model also agreed with the observations of the default sugar supply after fitting the selected parameters with GA (Table 2; Fig. 7B). After model implementation of the observed phloem sucrose concentration in the shading treatment, being 23% less than the default, exactly the same 36% reduction in fruit weight was predicted as had been observed. This simulated growth reduction was induced by a decrease in sugar concentrations, mainly in the vacuoles, and was reflected by the hexose tissue concentrations that were ∼13% decreased. This decrease was of the same order of magnitude as the observed 17% (see Table 2). When we incorporated the delta-pH time course as fitted for the shading treatment by Chen *et al*. (2021), we predicted a 30% reduction in fruit weight, which agreed less with observations, whereas predicted hexose content was similar to our simulation. The cell division rate was assumed to be reduced proportionally to the phloem sucrose decrease, as for cv. Moneyberg, but no observations were present for confirmation.

### Time course of hexose concentrations in the various treatments

Modelled hexose concentrations in the course of fruit growth of cv. Cappricia and cv. Brioso were slightly decreasing with time and agreed reasonably well with those reported by Okello *et al*. (2015) (Fig. 6). For cv. Moneymaker, the modelled hexose concentrations agreed well with the observed values that were consistently lower than for the other cultivars. Moreover, the drop in hexose concentration in the shading treatment that started at ∼30 DAA was correctly simulated (Chen *et al*., 2021). The good results for cv. Moneymaker led to low values for MAE and RRMSE (Table 4). In general, simulated concentrations of hexoses never exceeded ∼150 μmol g^−1^ FW tissue because any excess was immediately consumed in the metabolic pathway, where enzymatic processes were not rate-limiting. Any decrease in source supply of sucrose led to lower sugar concentrations and a growth decrease because of a lower osmotic sink for water, and thus lower water and concomitant mass flow, and a lower diffusive flow because of a lower source concentration. Any increase in sugar supply resulted in enhanced growth, as long as the potential growth according to maximum cell expansion was not yet reached. These interactions between concentration and expansion resulted in relatively stable hexose concentrations that were also observed in experiments, where lower fruit load, resulting in higher sugar availability, did not lead to higher hexose concentrations but did generate higher growth rates in the range of source-limited climate conditions (De Koning, 1994; Fanwoua *et al*., 2012a).

### Model sensitivity to selected sucrose uptake approach, vacuolar pH, and sucrose carrier

The computations with different ways of modelling sucrose uptake showed significant differences in modelled hexose concentrations (Fig. 8). When the mass flow was not constrained with a maximum level relative to the original model, hexose concentrations were consequently too high. In contrast, the original settings on active hexose uptake led to underestimation of hexose concentration, and were changed in the applied model (see model description section). All other computations showed a downward trend of hexose concentration that, below a certain threshold, may impede fruit growth. A change in the pH difference between the cytoplasm and acid vacuole of ±1 resulted in significant alterations of the hexose concentration in the fruit (ranging from −44% to +31%), as well as a change in final fresh fruit weight (ranging from −10% to +17%) (Fig. 9). A change in the transport capacity of the vacuolar sucrose carrier *V*_maxSUC_ was also important for the resulting hexose concentration in the fruit and the fresh fruit weight, both decreasing by 11% when halving *V*_maxSUC_ and increasing by 7% when doubling *V*_maxSUC_. Surprisingly, influx of sucrose was not altered per gram fresh weight, although water import from phloem—and thus mass flow of sucrose—changed with −8% and +5% for halving or doubling *V*_maxSUC_, respectively, because of the changed hexose concentration and its effect on the osmolality of the fruit tissue.

**Fig. 8.**
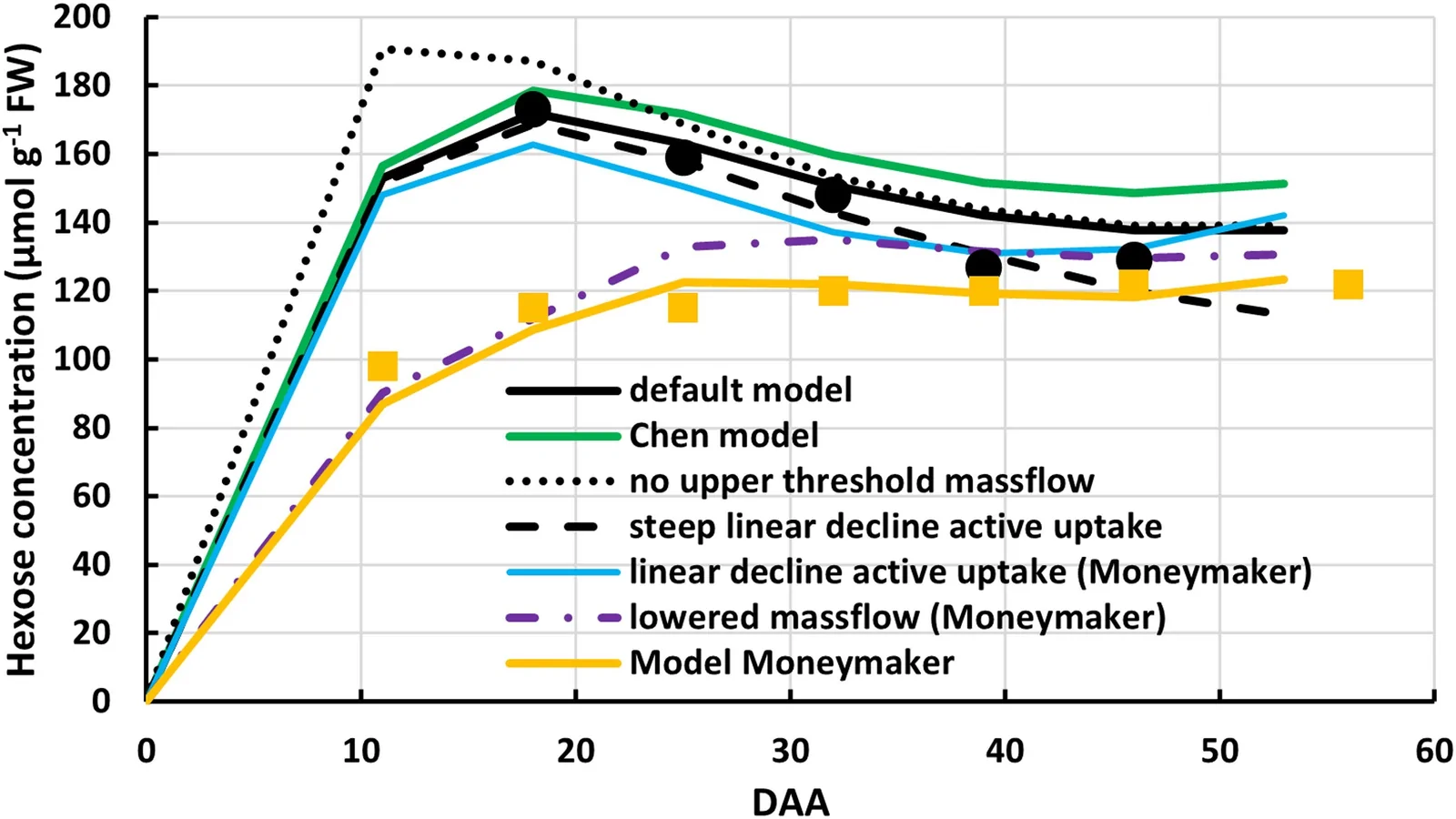
Sensitivity of model output to the selected model approach. Model output for the control treatment, using the default calibrated settings and three alternative approaches (cv. Cappricia), and using the default calibrated settings and two alternative approaches (cv. Moneymaker). Circles: observed hexose levels for cv. Cappricia; squares: observed hexose levels for cv. Moneymaker. The approach of a steep linear decline used the default maximum at start of growth and reached zero active flow at 46 days after anthesis (DAA). Values used for thresholds for mass flow and decay rate of active uptake are given in Supplementary Table S4B. Best fit for Cappricia was realized for the approach of a decay rate of active uptake as a function of the solute reflection coefficient (‘default’ with black line).

**Fig. 9.**
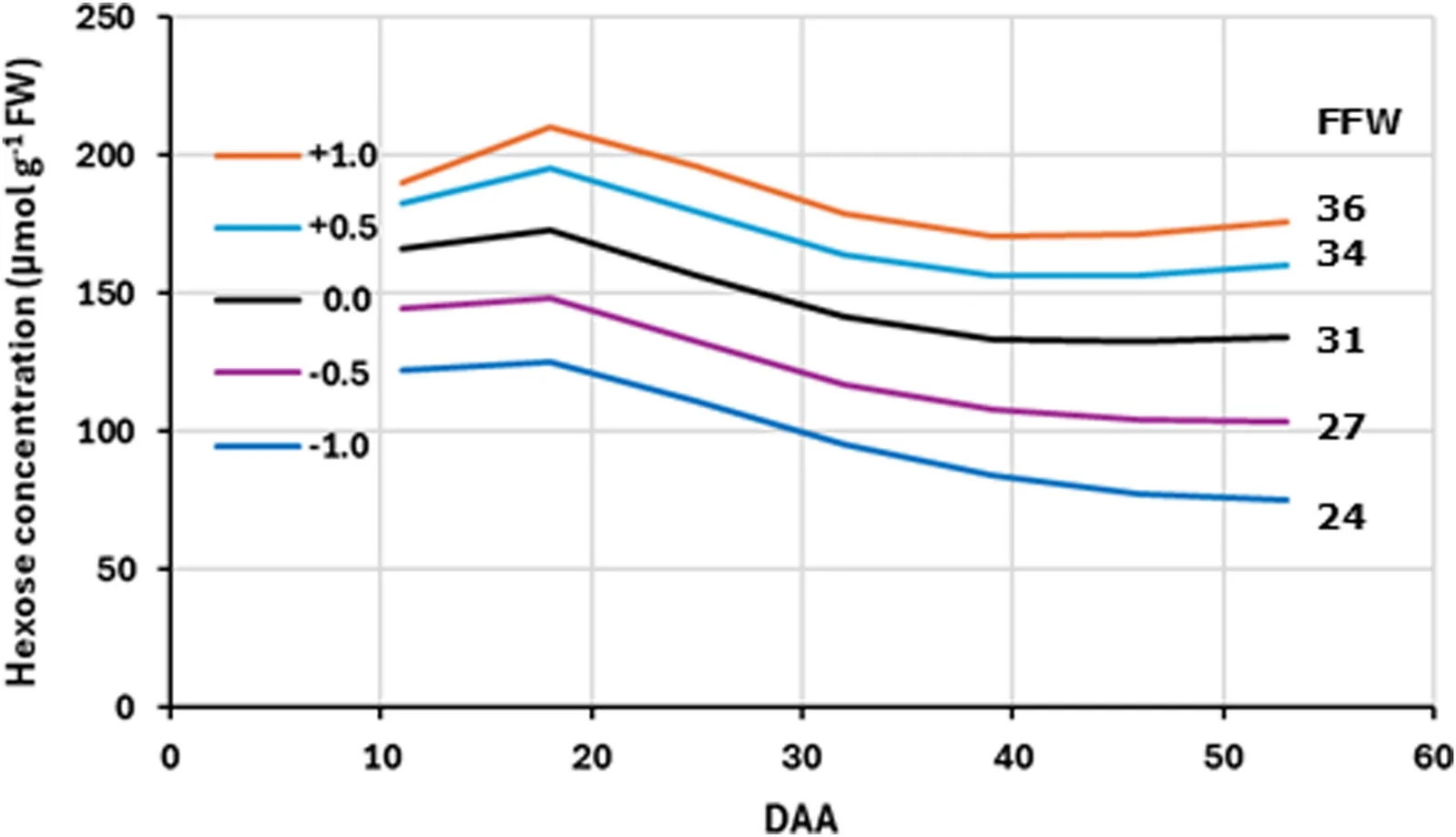
Sensitivity of modelled time course of hexose concentrations (in µmol hexose equivalents g^−1^ FW) and resulting fruit fresh weight (FFW in g). Model input was varied from a lower (min. −1) to a higher (max. +1) pH difference (delta-pH) than the mean of 2.9 between the neutral cytoplasm and acid vacuole for cv. Brioso.

### Modelled scenarios

The combination of increased temperature and a fruit load decrease to two fruits per truss—simulated by a 19% increase of the sucrose concentration in the phloem (*C*_p_) as shown for the fruit load trial above—resulted in unconstrained growth at 21 °C. Even at a *C*_p_ increase of 15%, the potential fruit fresh weight of 101 g could be reached for 21 °C, yet the potential weight of 141 g calculated with the fitted Richards parameters for 25 °C was only reached with a 45% increase.

The first dataset of cv. Moneyberg demonstrated that lowering fruit load resulted in a dramatic 42% increase in fruit weight (Table 2). With the model settings tuned for that experiment, a simulation of a rise in temperature to 25 °C showed that lowering fruit load led to an increase in fruit weight for cv. Moneyberg from 44 g to 60 g, an ∼36% increase, yet not up to the model-based potential weight of 68 g. Thus, both cultivars indicated that the potential weight at 25 °C cannot be met by a fruit load reduction to two fruits per truss. The model can also express these results on a cell basis: in cv. Moneyberg the final cell weight reached its potential size (23×10^–6^ g) at low fruit load at 21 °C. In contrast, at 25 °C it only reached its potential cell size (19×10^–6^ g) when phloem sucrose concentration was further increased from 1.19 (the supposed concentration at a load of two fruits per truss) to 1.27 times the default.

In the scenario in which the active uptake of hexose was fully blocked, total sugar uptake was reduced 50%, and a drastic decrease in the fruit fresh weight (−21%) and the hexose concentration (−38%) was found, while the sucrose uptake by mass and passive flow was negligibly increased (+1%), all relative to the default situation. The complete lack of hexose influx also led to a lower hydraulic conductance, since fruit growth and subsequent construction of vascular area to support water and symplastic sucrose inflow were hampered from the onset of fruit growth onwards. A 50% reduction of hexose influx led to a 12, 25, and 20% reduction of fruit fresh weight, hexose plus sucrose uptake, and hexose concentration, respectively.

The scenario in which the activity of the Susy (sucrose synthase) enzyme was fully silenced showed a mild increase of the hexose content from 116 µmol g^−1^ FW to 120 µmol g^−1^ FW, with 46 µmol fructose g^−1^ FW and 48.5 µmol fructose g^−1^ FW, for default and silenced conditions, respectively. These results confirm the finding that silencing Susy makes the fruit taste sweeter (i.e. increases its fructose content; Fernie and Martinez-Rivas, 2025).

### Conclusions

The mechanisms in which temperature plays an important role, such as determining sugar status and growth of tomato fruit, were conceptually incorporated via an existing physiological fruit model. The calibration of the temperature coefficients was not trivial since large differences in temperature response between cultivars were observed. The translation of temperature-driven, potential cell expansion into sink strength, confronted with source supply modelled from sugar dynamics, facilitated the prediction of temperature and sugar-driven cell division, cell expansion, sugar contents, and, ultimately, fruit growth.

Sugar limitation mostly depended on sugar supply (i.e. sugar concentration at the enzyme location; incorporated in the model via Michaelis–Menten equations), rather than on a limited pull strength by the sink for sugars. This supply is governed by the flow rate and the activity of the sucrose and hexose carriers of the tonoplast, importantly influenced by vacuolar acidity as shown in the sensitivity analysis, whereas the enzymic conversions hardly affected the pulling sink strength. The latter two conclusions were already drawn by Beauvoit *et al*. (2014). Surprisingly, the model showed that active uptake of apoplastic hexose contributes about half of the sugar supply, although the knockout of hexose carriers might stimulate other ways of hexose influx (e.g. diffusion) that were not incorporated in the model. The simulated fruit weight reductions of 12–21% due to a 50% reduction in hexose import were within the range reported by McCurdy *et al*. (2010).

## Discussion

This modelling study gives a first, provisional insight into the multiple growth processes that explain fruit weight and sugar contents of specific tomato cultivars. Although the dataset used to calibrate the model is small, it shows the growth mechanisms that occur under different temperature and source supply conditions. The use of extensibility as a function of the rate of potential cell expansion is a newly added feature to the existing biophysical model (Fishman and Génard, 1998; Liu *et al*., 2007), in which an empirical, time-dependent, but not temperature-dependent extensibility at fruit level was used. Our parameters and potential growth could also be interpreted as a measure of endoreduplication—the potential of the cell to reach a maximum ploidy with corresponding potential size according to the karyoplasmic ratio (Chevalier *et al*., 2014). Baldazzi *et al*. (2019) translated the ploidy level of the cell to a measure for cell wall extensibility to be applied in the biophysical model we used. They calibrated their endoreduplication model with observed cell size increment in time, as we did for the Richards parameters for potential cell size growth in thermal time. Ultimately, their model and ours use the same mechanisms to compute the actual cell expansion: the result of the interaction of sink demand (potential ploidy or our potential size, as well as cell-age-related biophysical properties) and actual resource availability at fruit level. The explanatory power of the model would increase if any insights were available on the effect of growth conditions on endoreduplication and associated cell wall extensibility as suggested by Apri *et al*. (2014).

The temperature response was approximated by using an activity coefficient for metabolic and developmental process rates and a thermal time approach for fruit ontogeny. Since we had data of only two temperature setpoints, any conclusion to predict the response at another temperature was not possible, since inter- or extrapolation requires knowledge of the shape of the response curve. Commonly, a Q_10_ for temperature response is used, which is not fundamentally different from our activity or Arrhenius coefficient (Mundim *et al*., 2020). However, determining the Q_10_ values should involve experiments on individual physiological processes at different temperatures, and by determining the activation energy per enzyme.

The observed reduction of cell weight at higher temperature was similar in relative terms for the cultivars used (Fig. 5). In the literature, most studies of temperature effects on fruit cell dynamics conclude that reduced cell volume is the mechanism to explain smaller fruits when heated, because the cell expansion duration is shortened to a greater degree than the cell expansion rate is increased (De Koning, 1994; Adams *et al*., 2001; Bertin, 2005; Fanwoua *et al*., 2012b; Okello *et al*., 2015). For temperature effects on cell division, there is less consensus, showing conflicting results between the referred studies. Interestingly, for cell division—and ultimately fruit weight—the temperature responses observed, and the associated activity coefficients showed large differences among cultivars (Table 3). Cvs Moneyberg, Cappricia, and Brioso showed a decreased, equal, or increased cell number, respectively, in response to a temperature rise of 4 °C (Table 2). Only the decreased cell number of cv. Moneyberg agrees with observations reported on cv. Moneymaker where cell number was reduced when heated from 20 °C to 25 °C, which was explained by a reduced period of cell division rather than a reduced rate of cell division (Bertin, 2005). Surprisingly, another cv. Moneyberg experiment in the same study showed a less negative response to temperature. The differences in cell division between cultivars suggest a wide genetic variation, contrary to the review by Van der Ploeg and Heuvelink (2005) where differences in temperature were shown to be rather similar between commercial cultivars, opposite to differences between wild tomato species.

In contrast to Q_10_, the use of thermal time for development of cells and their constituents such as membrane conductivity implies a linear relationship with temperature. For the used dataset of only two temperatures, this is not problematic, because this is the case as both temperatures were well above the base temperature and well below the optimum temperature for fruit growth rate. Yet, the question remains of whether the used thermal time is valid for other temperatures for such processes. For organ growth, these equations are correct and universally used (e.g. De Koning, 1994; Marcelis *et al*., 2009).

The observed sugar concentrations in fruits of cvs Moneyberg, Cappricia, and Brioso we used for calibration hardly differed between temperature treatments (Table 2; Fig. 6). Since cell division rates did differ by temperature-enhanced rate and development, the fruit seemed to balance the sugar concentrations to maintain homeostasis. Because we used a rather limited dataset, we cannot generalize these findings to other cultivars, since the lack of increase in sugar concentration to temperature contradicts more general findings that Brix and dry matter content are higher at higher temperatures (e.g. van der Ploeg and Heuvelink, 2005; Xiaoa *et al*., 2018). If sucrose would have increased, we would expect an increase in cell division rate, as was shown by Baldet *et al*. (2006), who observed an increased cell number resulting from sucrose-enhanced expression of cell proliferation-related genes. Since this mechanism occurred very early during fruit development (i.e. just after the flowers in the tomato truss appeared and before fertilization), it is debatable whether this also plays a role further in fruit development. Work on transcriptional and metabolic regulation of fruit growth showed that knowledge is still lacking to build a complete, multiscale, physiological fruit model (e.g. Carrari and Fernie, 2006; Niklas and Kutschera, 2012; Mauxion *et al*., 2021; Xu *et al*., 2025).

Since both cell number and cell size are simulated separately, the model is able to distinguish between the effects on fruit sink strength of either cell number or cell expansion. Interestingly, the model showed that a reduced cell number can, to a large extent, be compensated by increased cell expansion as long as water and sugars are available. On the other hand, when there were more but smaller cells simulated, the model can explain the reasons for the ratio of actual versus potential cell size by applying the underlying biophysical and metabolic mechanisms. Contrary to this ability to explain cell expansion, the model lacked physiological mechanisms that underly cell division. In future modelling, this could be worthwhile to include, as cell numbers may affect fruit taste, and cell division is one of the determinants of fruit sink strength (Ho, 1996). A good approach might be the explicit modelling of the different cell age classes using their age-dependent proliferation and expansion rates (Bertin *et al*., 2007; Fanwoua *et al*., 2013). Also the effect of sugars on the cell division rate could be made mechanistic, replacing our descriptive reduction factor based on phloem concentration. According to Biais *et al*. (2014), sugar effects on cell division were supported by, for example, data of Jin *et al*. (2009), explaining the sensitivity of fruit growth to carbon availability during cell division, and carbon starvation leading to fruit abortion (Ruan *et al*., 2012). In the literature, fructokinase is claimed to play a crucial role in floral initiation and abortion (Carrari and Fernie, 2006). Yet, our model could fully explain the growth differences under shading and higher fruit load by a decrease of phloem sucrose concentration resulting in reduced turgor-driven growth, without using a signalling mechanism (Fig. 7). The sugar influence on expansion could already be apparent in the division phase, as suggested by Ruan *et al*. (2012): then, hexose availability is most important to support the high glycolytic flux required by high rates of ATP-consuming growth processes, enabled by a fast influx of sucrose in vacuoles to start expansion. During cell expansion, hexose concentrations generally stay quite stable, which is not trivial to model given all the underlying processes. According to Biais *et al*. (2014), the apparent stability of hexoses may simply result from an adjustment of cell expansion, because fruits of shaded and water-limited plants were smaller. They claimed that in the case of shaded plants, sugar supply to the fruits may limit cell expansion, whereas under water limitation, at equal sugar concentrations, turgor potential would be less because of a decreased water potential. Our model system supports their vision that the mechanisms that determine vacuolar hexose concentrations play a major role in cell expansion.

As for the modelled scenario on unconstrained growth at 25 °C, the potential growth was reached for Cappricia—similar to Moneyberg—at a load of two fruits per truss, as is commonly used to determine potential size for averaged-sized tomato cultivars (Fanwoua *et al*., 2012a). On the basis of the Richards function for sink strength, a higher temperature would—at the default fruit load—lead to a smaller fruit because the source supply is constrained due to a shorter ripening period. Yet the increased sugar availability at lower fruit load can meet the higher peaks in sugar demand.

A model improvement on the simulation of sugar effects on cell division could also use the promising approach by Apri *et al*. (2014) in which the genetic regulatory network of transcription factors of cell cycle and expansion genes depends on sugar availability and sucrose signalling. Incorporating such a model will provide another challenge to connect the different spatial and temporal scales. Moreover, direct observations of cellular dynamics of different levels of sugar and hormone concentrations are still scarce, and this limits model parameterization.

The kinetic model of Chen *et al.*/Beauvoit *et al.* used was intended to simulate all relevant sugar sinks of the fruit, yet some important sugar pathways are still missing: (i) storage of G1P in starch by the action of AGPase; (ii) the use of G6P for the oxidative pentose phosphate pathway (OPPP); and (iii) all the sinks present in glycolysis, including acid metabolism and respiration. The model presented here misses the important contribution of organic acids—malate and citrate—in the storage of carbon and their effects on pH and concentration of soluble solids. This could be solved by incorporating an acid metabolism model (e.g. those by Lobit *et al*., 2003, 2006), but this was beyond the scope of the current modelling study. In addition, to further detail the fate of the carbon compounds in the fruit, existing data of observed fruit composition and carbon partitioning during fruit development could be used to increase the reliability of the model (Colombié *et al*., 2015). Recent work indicated that this integration of multiple technologies with mechanistic analysis such as experimental, analytical, and modelling work is the most promising way to explain total soluble contents as a major indicator of tomato fruit quality (Xu *et al*., 2025).

## Supplementary Material

erag331_Supplementary_Data

## Data Availability

The primary findings are presented within the article or in the related Supplementary data. Additional information and data can be made available by the corresponding author (PV) upon reasonable request.

## Supplementary data

The following supplementary data are available at *JXB* online.

Table S1. Rate equations of the integrative model.

Table S2. Ordinary differential equations (ODEs) for the integrative model.

Table S3. Summary of the inputs, outputs, and variables of the integrative model.

Table S4. Summary of the parameters whose values are from experimental measurements or the literature, and estimated parameters by model calibration.

Table S5. Thermal time-dependent functions of enzyme capacities derived for cv. ‘Moneymaker’.

Table S6. Fruit subcellular volume fractions, nucleotide concentrations, and vacuolar ΔpH.

Supplementary references

Protocol S1. Biophysical relationships on sugar and water flows in the fruit growth model.

Protocol S2. MATLAB code of the integrative model on sugar kinetics and fruit growth.
