# Supplementary material for: A sugar flow model predicts cell dynamics, weight, and quality of tomato at varying sink–source ratios and temperatures
Source: J Exp Bot. 2026 Jul 4;77(19):6713–28. doi: 10.1093/jxb/erag331 (PMC13672990; doi:10.1093/jxb/erag331)
Supplement: Supplementary file 1 [file erag331_supplementary_data.pdf]

Journal of Experimental Botany Supplementary information

Article title: A sugar flow model predicts cell dynamics, weight, and quality of tomato at varying sink-source ratios and temperatures

Authors: Pieter de Visser, Jaap Molenaar, Paul Struik

Article acceptance date: 10 June 2026

Contents:

Table S1. Rate equations of the integrative model.

Table S2. Ordinary differential equations (ODEs) for the integrative model.

Table S3a. Summary of the inputs and outputs of the integrative model.

Table S3b. Summary of the variables of the integrative model.

Table S4a. Summary of the parameters whose values are taken from experimental measurements or literature.

Table S4b. Values of the best parameter set per cultivar for the integrative model estimated by genetic algorithm and post-GA tuning by fitting the model to the observations indicated.

Table S5. Thermal time dependent functions of enzyme capacities derived for the tomato cultivar 'Moneymaker'.

Table S6. Fruit subcellular volume fractions, nucleotide concentrations, and vacuolar ΔpH.

Supplementary references

Supplementary protocol S1. Biophysical relationships on sugar and water flows in the fruit growth model.

Supplementary protocol S2. MATLAB code of the integrative model on sugar kinetics and fruit growth.

Table S1. Rate equations of the integrative model.

Symplasmic sucrose uptake:

$$V0 = \frac{\left(U_{mass} + U_{passive}\right)/(342.3 * 10^6)}{S + W}$$

Apoplasmic hexose uptake:

$$V1 = \frac{U_{active}/(342.3 * 2 * 10^6)}{S + W}$$

Glucokinase (GK):

$$V2 = \frac{60 * \ V_{maxGK} \ * \ \frac{Glccyt}{K_{m2Glc}} \ * \ \frac{ATP}{K_{m2ATP}}}{\left(1 + \frac{ATP}{K_{m2atp}}\right) * \left(1 + \ \frac{Glccyt}{K_{m2Glc}} + \frac{Frucyt}{K_{m2GFru}} + \ \frac{G6P}{K_{i2G6P}} + \ \frac{F6P}{K_{i2F6P}}\right)}$$

Fructokinase (FK):

$$V3 = \frac{60 * \ V_{maxFK} \ * \ Frucyt * ATP}{K_{m3ATP} * Frucyt + \ K_{m3Fru} * ATP + \ Frucyt * ATP}$$

Sucrose-6-phosphate synthase (SPS):

$$V4 = \frac{60 * \ V_{maxSPS} \ * (F6P * UDPGlc - \frac{S6P * UDP}{K_{eq}}}{T1 + \ T2}$$

with:

$$T1 = \ F6P * UDPGlc * \left(1 + \ \frac{S6P}{K_{i4S6P}}\right) + \ K_{m4S6P} * \left(1 + \frac{Pi}{K_{i4P}}\right) * (UDPGlc + \ K_{i4UDPGlc}) + K_{m4UDP} * F6P$$

$$T2 = \ \frac{r_{VfVrSPS}}{K_{eq4}} * \ [S6P * K_{m4UDP} \ * \left(1 + \ \frac{UDPGlc}{K_{i\$UDPGlc}}\right) + UDP\left(K_{m4S6P}\left(1 + \qquad \frac{K_{m4UDPGlc} * F6P}{K_{i4UDPGlc} * K_{m4F6P}\left(1 + \frac{Pi}{K_{i4Pi}}\right)}\right) + S6P\left(1 + \frac{F6P}{K_{i4F6P}}\right)\right)]$$

Sucrose-6-phosphatase (SPase):

$$V5 = \frac{60 * V_{maxSPase} * S6P}{K_{m5S6P} + S6P}$$

Sucrose synthase (Susy):

$$V6 = \frac{-60 * \ V_{maxSusy} * (Succyt * UDP - \ \frac{Frucyt * UDPGlc}{K_{eq6}})}{T3 + T4}$$

with:

$$T3 = Succyt * UDP * \left(1 + \ \frac{Frucyt}{K_{i6Fru}}\right) + \ K_{m6Suc} * (UDP + K_{i6UDP}) + \ K_{m6UDP} * Succyt$$

$$T4 = \ \frac{r_{VfVrSusy}}{K_{eq6}} * [Frucyt * \ K_{m6UDPGk} * \left(1 + \frac{UDP}{K_{i6UDP}}\right) + UDPGlc * \{K_{m6Fru}\left(1 + \frac{K_{m6UDP} * Succyt}{K_{i6UDP} * K_{m6Suc}}\right) + Frucyt * \left(1 + \frac{Succyt}{K_{i6Suc}}\right)\}]$$

Neutral invertase (NI):

$$V7 = \frac{60 * V_{maxNI}}{1 + \frac{Glccyt}{K_{i7Glc}}} * \frac{Succyt}{K_{m7Suc} * \left(1 + \frac{Frucyt}{K_{i7Fru}}\right) + Succyt}$$

ATP-dependent phosphofructokinase (PFK):

$$V8 = \frac{60 * V_{maxPFK} * (F6P/F6P_{hal})^{h_{PFK}} * (ATP/ATP_{half})^{h_{PFK}}}{T5 + T6 + T7}$$

with:

$$T5 = \frac{1 + (ATP/ModATP_{half})^{h_{PFK}}}{1 + sigma^{4xh_{PFK}}(ATP/ModATP_{hal})^{h_{PFK}}}$$

$$T6 = \frac{\left[(F6P/F6P_{hal})^{h_{PFK}} + (ATP/ATP_{half})^{h_{PFK}}\right] * [1 + sigma^{2xh_{PFK}}(ATP/ATP_{half})^{h_{PFK}}]}{1 + sigma^{4xh_{PFK}}(ATP/ATP_{half})^{h_{PFK}}}$$

$$T7 = \left(\frac{F6P}{F6P_{half}}\right)^{h_{PFK}} * \left(\frac{ATP}{ATP_{half}}\right)^{h_{PFK}}$$

PPi-dependent phosphofructokinase (PFP):

$$V9 = \frac{60 * V_{maxPFP}}{K_{m9F6P} * K_{m9PPi}} * \frac{F6P * PPi - FBP * Pi/_{eq9}}{(1 + F6P/K_{m9F6P} + FBP/K_{m9FBP}) * (1 + PPi/K_{m9PPi} + Pi/K_{m9Pi})}$$

Vacuolar sucrose carrier:

$$V10 = \frac{60 * V_{maxSUC} * (Succyt/K_{m10Suc} - Sucvac/K_{m10Sucvac})}{(1 + Succyt/K_{m10Suc} + Sucvac/K_{m10Sucvac})}$$

Vacuolar acid invertase (AI):

$$V11 = \frac{60 * V_{maxAI}}{1 + Glcvac/K_{i11Glcvac}} * \frac{Sucvac}{K_{m11Sucvac} * (1 + Fruvac/K_{i11Fruvac}) + Sucvac}$$

Sucrose storage:

$$V12 = Sucvac * \frac{dS/dt + dW/dt}{S + W}$$

Glucose storage:

$$V13 = Glcvac * \frac{dS/dt + dW/dt}{S + W}$$

Fructose storage:

$$V14 = Fruvac * \frac{dS/dt + dW/dt}{S + W}$$

Vacuolar glucose carrier:

$$V15 = \frac{60 * V_{maxHEX} * [Glccyt/(K_{m15Glc} + Frucyt) - Glcvac/(K_{m15Glcvac} + Fruvac)]}{1 + Glccyt/(K_{m15Glc} + Frucyt) + Glcvac/(K_{m15Glcvac} + Fruvac)}$$

Vacuolar fructose carrier: $$V16 = \frac{60 * V_{maxHEX} * [Frucyt/(K_{m16F} + Glccyt) - (Fruvac/(K_{m16Fr} + Glcvac)]}{1 + Frucyt/(K_{m16Fru} + Glccyt) + Fruvac/(K_{m16Fruvac} + Glcvac)}$$
Phosphoglucose isomerase (PGI): $$V17 = \frac{60 * V_{maxPGI} * \left(\frac{G6P}{K_{m17G6P}} - \frac{1}{r_{VfVrPGI}} * \frac{F6P}{K_{m17F6}}\right)}{1 + G6P/K_{m17G6P} + F6P/K_{m17F6P}}$$
Phosphoglucomutase (PGM): $$V18 = \frac{60 * V_{maxPGM} * \left(\frac{G6P}{K_{m18G6P}} - \frac{1}{r_{VfVrPGM}} * \frac{G1P}{K_{m18G1P}}\right)}{1 + G6P/K_{m18G6P} + G1P/K_{m18G1P}}$$
UDP-glucopyrophosphorylase (UGPase): $$V19 = \frac{60 * V_{maxUGPase} * \left(\frac{UTP * G1P}{K_{m19UTP} * K_{m19G1}} - \frac{1}{r_{VfVrUGPase}} * \frac{UDPGlc * PPi}{K_{m19UDPGlc} * K_{m19PP}}\right)}{(1 + UDPGlc/K_{m19UDPGlc} + UTP/K_{m19UTP}) * (1 + PPi/K_{m19PPi} + G1P/K_{m19G1P})}$$
Aldolase (ALD): $$V20 = \frac{60 * V_{maxALD} * FBP}{K_{m20FBP} + FBP}$$
Starch storage: $$V21 = Starch * \frac{dS/dt + dW/dt}{S + W}$$
Cell wall storage: $$V22 = CellWall * \frac{dS/dt + dW/dt}{S + W}$$
G6P/Pi translocase (GPT): $$V23 = \frac{60 * V_{maxGPT}}{K_{m23G6} * K_{m23Piplast}} * T8$$ with: $$T8 = \frac{G6P * Piplast - G6Pplast * Pi}{(1 + G6P/K_{m23G6P} + G6Pplast/K_{m23G6Pplast}) * (1 + Piplast/K_{m23Piplast} + Pi/K_{m23Pi})}$$
Fru-1,6-bisphosphatase (FBPase): $$V24 = \frac{60 * V_{maxFBPase} * FBP}{K_{m24FBP} + FBP}$$
Starch synthesis: $$V25s = G6Pplast * \frac{k_{ss}}{1 + \exp((t - u_{ss})/t_{ss})}$$
Starch degradation: $$V25d = k_{ds} * Starch$$
Cell wall synthesis: $$V26s = UDPGlc * \frac{k_{sw}}{1 + \exp((t - u_{sw})/t_{sw})}$$

Cell wall degradation:
$$V26d = k_{dw} * CellWall$$

Footnotes: Vi are reaction rates expressed in μmol g^{-1} FW h^{-1}. K_m and K_i values are expressed in mM. The capacities of enzymes and transporters (V_{max}) are expressed in μmol g^{-1} FW min^{-1}. Concentrations applied in the kinetic equations are expressed in mM, while those applied in the equations describing the storage of sugars, starch, and cell wall as well as the synthesis and degradation of starch and cell wall are expressed in μmol g^{-1} FW. The conversion of concentration from mM in solution to μmol per gram fresh weight uses the volume fraction of vacuole (Vol_{vac}), cytoplasm (Vol_{cyt}) and plastid (Vol_{plast}) (see Table S6).

Table S2. Ordinary differential equations (ODEs) for the integrative model.

Cytoplasmic fructose	dFrucyt/dt = V1/2+V7-V3-V6-V16
Cytoplasmic glucose	dGlucyt/dt = V1/2+V7-V2-V15
Cytoplasmic sucrose	dSuccyt/dt = V0-V10+V5+V6-V7
Vacuolar fructose	dFruvac/dt = V16+V11-V14
Vacuolar glucose	dGlcvac/dt = V15+V11-V13
Vacuolar sucrose	dSucvac/dt = V10-V11-V12
S6P	dS6Pdt/dt = V4-V5
Cytoplasmic G6P	dGLC6P/dt = V2-V18-V17-V23
Plastidial G6P	dGLC6Pplast/dt = V23 – V25s
G1P	dG1P/dt = V18-V19+V25d
F6P	dFRU6P/dt = V3+V17+V24-V4-V8-V9
UDPGlc	dUDPGLC/dt = V19-V6-V4-V26s+V26d
FBP	dFBP/dt = V8+V9-V24-V20-V27
Cellwall	dCellwall/dt = V26s-V26d-V22
Starch	dStarch/dt = V25s-V25d-V21
Dry weight	$dS/dt = U_{active} + U_{mass} + U_{passive} + R_f$
Water	$dW/dt = U_x + U_p - T_f$

Footnotes: Vi are reaction rates expressed in μmol g^{-1} FW h^{-1}. Metabolites are expressed in μmol g^{-1} FW. Dry weight (S) and water (W) of fruit are expressed in g. Water fluxes (U_x and U_p), transpiration (T_f), sugar uptake rate (U_{active}, U_{mass}, and $U_{passive}$), and respiration (R_f) are expressed in g h^{-1}.

Table S3a. Summary of the inputs and outputs of the integrative model

Symbol	Description	Unit
**Inputs**		
Cp	Sucrose concentration in phloem sap	g g^{-1}
Ψx	Xylem water potential in stem	MPa
Ha	Air relative humidity	–
T	Air temperature	°C
VmaxGK	Enzyme capacity of glucokinase (GK)	µmol g^{-1} FW min^{-1}
VmaxFK	Enzyme capacity of fructokinase (FK)	µmol g^{-1} FW min^{-1}
VmaxSPS	Enzyme capacity of sucrose-6-phosphate synthase (SPS)	µmol g^{-1} FW min^{-1}
VmaxSusy	Enzyme capacity of sucrose synthase (Susy)	µmol g^{-1} FW min^{-1}
VmaxNI	Enzyme capacity of neutral invertase (NI)	µmol g^{-1} FW min^{-1}
VmaxPFK	Enzyme capacity of ATP-dependent phosphofructokinase (PFK)	µmol g^{-1} FW min^{-1}
VmaxPFP	Enzyme capacity of PPi-dependent phosphofructokinase (PFP)	µmol g^{-1} FW min^{-1}
VmaxAI	Enzyme capacity of vacuolar acid invertase (AI)	µmol g^{-1} FW min^{-1}
VmaxPGI	Enzyme capacity of phosphoglucose isomerase (PGI)	µmol g^{-1} FW min^{-1}
VmaxPGM	Enzyme capacity of phosphoglucomutase (PGM)	µmol g^{-1} FW min^{-1}
VmaxUGPase	Enzyme capacity of UDP-glucopyrophosphorylase (UGPase)	µmol g^{-1} FW min^{-1}
VmaxALD	Enzyme capacity of fructose-1,6-bisphosphate aldolase (ALD)	µmol g^{-1} FW min^{-1}
VmaxFBPase	Enzyme capacity of fructose-1,6-bisphosphatase (FBPase)	µmol g^{-1} FW min^{-1}
$NRCells_{ini}$	Initial number of fruit cells of an average fruit	-
$Cellmass_{ini}$	Initial freshweight of the average fruit cell	g FW
**Outputs**		
S	Fruit dry weight	g
W	Fruit water amount	g
Cell number	Mean cell number	-
Cellmass	Mean cell mass	g FW
Frucyt	Fructose concentration in cytoplasm	µmol g^{-1} FW
Fruvac	Fructose concentration in vacuole	µmol g^{-1} FW
Glccyt	Glucose concentration in cytoplasm	µmol g^{-1} FW
Glcvac	Glucose concentration in vacuole	µmol g^{-1} FW
Succyt	Sucrose concentration in cytoplasm	µmol g^{-1} FW
Sucvac	Sucrose concentration in vacuole	µmol g^{-1} FW
S6P	Sucrose-6-phosphate concentration in cytoplasm	µmol g^{-1} FW
G6P	Glucose-6-phosphate concentration in cytoplasm	µmol g^{-1} FW
G6Pplast	Glucose-6-phosphate concentration in plastid	µmol g^{-1} FW
F6P	Fructose-6-phosphate concentration in cytoplasm	µmol g^{-1} FW
G1P	Glucose-1-phosphate concentration in cytoplasm	µmol g^{-1} FW

UDPGlc	UDP-glucose concentration in cytoplasm	µmol g^{-1} FW
FBP	Fructose-1,6-bisphosphate concentration in cytoplasm	µmol g^{-1} FW
Starch	Starch concentration	µmol glucose eq. g^{-1} FW
CellWall	Cell wall concentration	µmol glucose eq. g^{-1} FW
OrgRest	Concentration of the total of organic compounds other than carbohydrates (so, organic and amino acids, proteins, fats)	µmol glucose eq. g^{-1} FW

Footnote: FW, fresh weight; eq., equivalent.

Table S3b. Summary of the variables of the integrative model.

P_f	Fruit hydrostatic pressure	MPa
Π_f	Fruit osmotic pressure	MPa
P_p	Phloem hydrostatic pressure	MPa
Π_p	Phloem osmotic pressure	MPa
P_x	Xylem hydrostatic pressure	MPa
Π_x	Xylem osmotic pressure	MPa
A_f	Fruit surface area	cm^2
A_p	Membrane area of exchange between phloem vessels and the fruit compartment	cm^2
A_x	Membrane area of exchange between xylem vessels and the fruit compartment	cm^2
U_p	Water flux into fruit from phloem	$g\ h^{-1}$
U_x	Water flux into fruit from xylem	$g\ h^{-1}$
T_f	Fruit transpiration	$g\ h^{-1}$
Σ_p	Reflection coefficient of membrane to solute between phloem vessels and fruit compartment	–
V_{maxup}	Capacity of active sugar transport across the plasma membrane	g suc eq. g^{-1} DW h^{-1}
U_{active}	Active sugar uptake rate	$g\ h^{-1}$
U_{mass}	Sugar intake rate to fruit by mass flow in phloem	$g\ h^{-1}$
$U_{passive}$	Sugar intake rate to fruit by passive diffusion	$g\ h^{-1}$
R_f	Fruit respiration	$g\ h^{-1}$
Vol	Fruit volume	cm^3
Φ	Cell wall extensibility	$MPa^{-1}\ h^{-1}$
Cellnr	Cell number	$fruit^{-1}$
CR_{div}	Cell division rate	min^{-1}
Cellsize	Cell fresh weight	g FW $cell^{-1}$
RGR_{cell}	Relative growth rate of cell fresh weight	min^{-1}
Vol_{vac}	Vacuole volume fraction	–
Vol_{cyt}	Cytoplasm volume fraction	–
Vol_{plast}	Plastid volume fraction	–
ATP	ATP concentration in cytoplasm	µmol g^{-1} FW
ADP	ADP concentration in cytoplasm	µmol g^{-1} FW
UTP	UTP concentration in cytoplasm	µmol g^{-1} FW
UDP	UDP concentration in cytoplasm	µmol g^{-1} FW
Pi	Inorganic phosphate concentration in cytoplasm	µmol g^{-1} FW
Piplast	Inorganic phosphate concentration in plastid	µmol g^{-1} FW
PPi	Inorganic pyrophosphate concentration in cytoplasm	µmol g^{-1} FW
ΔpH	Difference of pH value between cytoplasm and vacuole	–
V0	Sucrose uptake rate by mass flow and passive diffusion in phloem	µmol g^{-1} FW h^{-1}
V1	Hexose uptake rate by active transport	µmol g^{-1} FW h^{-1}

V2	Reaction flux of glucokinase (GK)	μmol g^{-1} FW h^{-1}
V3	Reaction flux of fructokinase (FK)	μmol g^{-1} FW h^{-1}
V4	Reaction flux of sucrose-6-phosphate synthase (SPS)	μmol g^{-1} FW h^{-1}
V5	Reaction flux of sucrose-6-phosphatase (SPase)	μmol g^{-1} FW h^{-1}
V6	Reaction flux of sucrose synthase (Susy)	μmol g^{-1} FW h^{-1}
V7	Reaction flux of neutral invertase (NI)	μmol g^{-1} FW h^{-1}
V8	Reaction flux of ATP-dependent phosphofructokinase (PFK)	μmol g^{-1} FW h^{-1}
V9	Reaction flux of PPi-dependent phosphofructokinase (PFP)	μmol g^{-1} FW h^{-1}
V10	Sucrose transport flux of sucrose carrier on tonoplast	μmol g^{-1} FW h^{-1}
V11	Reaction flux of vacuole acid invertase (AI)	μmol g^{-1} FW h^{-1}
V12	Sucrose storage due to fruit expansion Glucose storage due to fruit expansion	μmol g^{-1} FW h^{-1}
V13	Fructose storage due to fruit expansion	μmol g^{-1} FW h^{-1}
V15	Glucose transport flux of hexose carrier on tonoplast	μmol g^{-1} FW h^{-1}
V16	Fructose transport flux of hexose carrier on tonoplast	μmol g^{-1} FW h^{-1}
V17	Reaction flux of phosphoglucose isomerase (PGI)	μmol g^{-1} FW h^{-1}
V18	Reaction flux of phosphoglucomutase (PGM)	μmol g^{-1} FW h^{-1}
V19	Reaction flux of UDP-glucopyrophosphorylase (UGPase)	μmol g^{-1} FW h^{-1}
V20	Reaction flux of fructose-1,6-bisphosphate aldolase (ALD)	μmol g^{-1} FW h^{-1}
V21	Starch storage due to fruit expansion	μmol g^{-1} FW h^{-1}
V22	Cell wall storage due to fruit expansion	μmol g^{-1} FW h^{-1}
V23	Glucose-6-phosphate transport flux of glucose-6-phosphate/Pi translocase	μmol g^{-1}FW h^{-1}
V24	Reaction flux of fructose-1,6-bisphosphatase (FBPase)	μmol g^{-1} FW h^{-1}
V25s	Starch synthesis rate	μmol glucose eq. g^{-1} FW h^{-1}
V25d	Starch degradation rate	μmol glucose eq. g^{-1} FW h^{-1}
V26s	Cell wall synthesis rate	μmol glucose eq. g^{-1} FW h^{-1}
V26d	Cell wall degradation rate	μmol glucose eq. g^{-1} FW h^{-1}
V_{rest}	Flux of monosaccharides remaining after glycolysis and respiration	μmol g^{-1} FW h^{-1}
V_{maxSUC}	Transport capacity of vacuolar sucrose carrier on tonoplast	μmol g^{-1} FW h^{-1}
V_{maxHEX}	Transport capacity of vacuolar hexose carrier on tonoplast	μmol g^{-1} FW h^{-1}
k_{syn_sta}	Starch biosynthesis rate constant	h
k_{syn_cw}	Cell wall biosynthesis rate constant	h

Footnote: FW, fresh weight; suc, sucrose; eq., equivalent.

Table S4a Summary of the parameters of the integrative model whose values are taken from experimental measurements or literature.

Symbol	Description	Value	Unit	Reference
**Fruit growth model**				
q_{m293}	Maintenance respiration coefficient	0.00042	g glucose g^{-1} DW hr^{-1}	Bertin and Heuvelink (1993)
q_g	Growth respiration coefficient	0.3	g glucose g^{-1} DW	Heuvelink (1995)
Q_{10}	Q_{10} for maintenance respiration	1.4	-	Bertin and Heuvelink (1993)
T_{base}	Base temperature for tomato fruit growth (cvs Cappricia and Brioso)	3	°C	Okello *et* al. (2014)
T_{base}	Base temperature for tomato fruit growth (cvs Moneyberg and Moneymaker)	5.7	°C	Adams *et al*. (2001)
$T_{sumRipe}$	Temperature sum fruit ripening cv. Cappricia	992	°Cd	Okello *et al*. (2014)
$T_{sumRipe}$	Temperature sum fruit ripening cv. Brioso	920	°Cd	Okello *et al*. (2014)
$T_{sumRipe}$	Temperature sum fruit ripening cvs Moneyberg and Moneymaker	881	°Cd	Fanwoua *et al*. (2012)
p_s	Phloem membrane permeability for sugar diffusion	$3.6*10^{-5}$	g cm^{-2} h^{-1}	Ruan and Patrick (1995)
Φ_{max}	Maximum cell wall extensibility	0.02	bar^{-1} h^{-1}	Cosgrove (1985)
Y	Threshold hydrostatic pressure for cell growth	1	bar	Grange (1995)
H_f	Relative humidity of air space in fruit	0.996	-	Fishman and Génard (1998)
ρ	Permeation coefficient of fruit surface to water vapor	$0.162*10^{-4}$	g m^{-2} hr^{-1} MPa^{-1}	De Swaef and Steppe (2010)
L_x	Xylem conductivity for water transport relative to phloem conductivity for water	0.22	-	Liu *et al*. (2007)
σ_x	Reflection coefficient of membrane to solute between xylem vascular tissue and fruit compartment	1	-	Fishman and Genard (1998)
π_{P0}	Osmotic pressure due to the solutes other than sugars in phloem	7	bar	Chen *et al*. (2021)
π_{F0}	Osmotic pressure due to the solutes other than sugars in fruit	3.5	bar	Chen *et al*. (2021)
r_w	g water produced per g respired glucose	0.6	–	
γ	Parameters relating fruit surface area to fresh weight	10.43	-	Chen *et al*. (2021)
η		0.533	-	Chen *et al*. (2021)

axp	Ratio of the area of exchange between vascular networks and the fruit compartment to the fruit surface area	0.0163	-	Chen *et al*. (2021)
τ_s	Parameter describing the variation of phloem membrane reflection coefficient to sugar with fruit age	$1.38*10^{-6}$	h^{-1}	Chen *et al*. (2021)
D_w	Density of water	1.0	g cm^{-3}	
D_s	Density of dry mass	1.6	g cm^{-3}	
M_w	Molecular weight of water	18.0	g mol^{-1}	
M_s	Molecular weight of sucrose	342.3	g mol^{-1}	
M_h	Molecular weight of hexose	180.2	g mol^{-1}	
R	Gas constant	83	cm^3 bar mol^{-1} K^{-1}	
K_m	Michaelis constant for active sugar uptake	0.08	g g^{-1}	Milner *et al*. (1995)
**Kinetic model**				
Km2Glc		0.07	mM	Values below from Beauvoit *et al*. (2014)
Km2Fru	Michaelis constant of GK for fructose	10	mM	unless indicated otherwise
Km2ATP	Michaelis constant of GK for ATP	0.25	mM	
Ki2G6P	Inhibition constant of GK for G6P	0.1	mM	
Ki2F6P	Inhibition constant of GK for F6P	10	mM	
Km3Fru	Michaelis constant of FK for fructose	0.028	mM	
Km3ATP	Michaelis constant of FK for ATP	0.14	mM	
rVfVrSPS	Ratio of forward to reverse activity for SPS	1.923	–	
Keq4	Equilibrium constant of SPS	10	–	
Km4F6P	Michaelis constant of SPS for F6P	0.6	mM	
Km4UDPGlc	Michaelis constant of SPS for UDPGlc	1.8	mM	
Km4UDP	Michaelis constant of SPS for UDP	0.3	mM	

Km4S6P Michaelis constant of SPS for S6P	0.1	mM
KiPi Inhibition constant of SPS for Pi	3	mM
Ki4F6P Inhibition constant of SPS for F6P	0.4	mM
Ki4UDPGlc Inhibition constant of SPS for UDPGlc	1.4	mM
Ki4S6P Inhibition constant of SPS for S6P	0.07	mM
VmaxSPase Enzyme capacity of sucrose-6-phosphatase (SPase)	0.125	µmol g^{-1} FW min^{-1}
Km5S6P Michaelis constant of Spase for S6P	0.1	mM
rVfVrSusy Ratio of forward to reverse activity for Susy	0.5	-
Keq6 Equilibrium constant of Susy	0.5	–
Km6Suc Michaelis constant of Susy for sucrose	35.9	mM
Km6UDP Michaelis constant of Susy for UDP	0.00191	mM
Km6UDPGlc Michaelis constant of Susy for UDPGlc	0.234	mM
Km6Fru Michaelis constant of Susy for fructose	6.49	mM
Ki6Suc Inhibition constant of Susy for sucrose	139	mM
Ki6Fru Inhibition constant of Susy for fructose	3.1	mM
Ki6UDP Inhibition constant of Susy for UDP	0.0871	mM
Km7Suc Michaelis constant of NI for sucrose	10	mM
Ki7Fru Inhibition constant of NI for fructose	15	mM
Ki7Glc Inhibition constant of NI for glucose	15	mM
F6Phalf Kinetic parameters for PFK	0.758	mM
ATPhalf	0.155	mM
h_PFK	2.3	–
ModATPhalf	2	mM
sigma	0.9	–

Keq9 Equilibrium constant for PFP	3.3	-
Km9FBP Michaelis constant of PFP for FBP	0.382	mM
Km9F6P Michaelis constant of PFP for F6P	1	mM
Km9PPi Michaelis constant of PFP for PPi	0.12	mM
Km9Pi Michaelis constant of PFP for Pi	0.51	mM
Km10Suc Michaelis constant of vacuolar sucrose carrier for sucrose in cytoplasm	1.2	mM
Km10Sucvac Michaelis constant of vacuolar sucrose carrier for sucrose in vacuole	Km10Suc*10ΔpH	mM
Km11Sucvac Michaelis constant of AI for sucrose	10	mM
Ki11Glcvac Inhibition constant of AI for glucose	26	mM
Ki11Fruvac Inhibition constant of AI for fructose	23.5	mM
Km15Glc Michaelis constant of vacuolar hexose carrier for glucose in cytoplasm	0.4	mM
Km15Glcvac Michaelis constant of vacuolar hexose carrier for glucose in vacuole	Km15Glc*10ΔpH	mM
Km16Fru Michaelis constant of vacuolar hexose carrier for fructose in cytoplasm	0.4	mM
Km16Fruvac Michaelis constant of vacuolar hexose carrier for fructose in vacuole	Km16Fru*10ΔpH	mM
rVfvrPGI Ratio of forward to reverse activity for PGI	0.565	-
Km17G6P Michaelis constant of PGI for G6P	0.31	mM
Km17F6P Michaelis constant of PGI for F6P	0.174	mM
rVfVrPGM Ratio of forward to reverse activity for PGM	0.0525	-
Km18G6P Michaelis constant of PGM for G6P	0.3	mM
Km18G1P Michaelis constant of PGM for G1P	0.3	mM
rVfVrUGPase Ratio of forward to reverse activity for UGPase	0.409	-
Km19UDPGlc Michaelis constant of UGPase for UDPGlc	0.5	mM
Km19UTP Michaelis constant of UGPase for UTP	0.15	mM
Km19PPi Michaelis constant of UGPase for PPi	0.15	mM

Km19G1P	Michaelis constant of UGPase for G1P	0.5	mM
Km20FBP	Michaelis constant of ALD	0.015	mM
VmaxGPT	Transport capacity of G6P/Pi translocase between plastids and cytoplasm	0.206	µmol g^{-1} FW min^{-1}
Km23G6P	Michaelis constant of G6P/Pi translocase for G6P in cytoplasm	0.6	mM
Km23Pi	Michaelis constant of G6P/Pi translocase for Pi in cytoplasm	1.1	mM
Km23G6Pplast	Michaelis constant of G6P/Pi translocase for G6P in plastid	0.6	mM
Km23Piplast	Michaelis constant of G6P/Pi translocase for Pi in plastid	1.1	mM
r_Pi	Plastid-to-cytoplasm Pi concentration ratio	30	-
Km24FBP	Michaelis constant of FBPase for FBP	0.1	mM Values below from Chen *et al*. (2021)
$a_{max}SUC$	Parameters describing the variation of transport capacity of tonoplastic sucrose carrier during fruit development ($V_{max}SUC$)	0.0326	µmol g^{-1} FW min^{-1}
$b_{max}SUC$		0.008574	h
$c_{max}SUC$		1209.26	h^{-1}
$d_{max}SUC$		0.001714	h
$a_{max}HEX$	Parameters describing the variation of transport capacity of tonoplastic hexose carrier during fruit development (VmaxHEX)	0.0344	µmol g^{-1} FW min^{-1}
$b_{max}HEX$		0.0018744	h^{-1}
k_{ds}	Starch degradation rate constant	0.0187	h^{-1}
k_{SS}	Parameters describing the variation of starch biosynthesis rate constant during fruit development (k_{syn_sta})	2.386	h^{-1}
u_{SS}		768.72	h
t_{SS}		166.32	h
a_{dw}	Cell wall degradation rate constant	0.00306	h^{-1}
k_{sw}	Parameters describing the variation of cell wall biosynthesis rate constant during fruit development (k_{syn_cw})	110.47	h^{-1}
u_{sw}		680.64	h
t_{sw}		496.8	h

Table S4b. Values of the best parameter set per cultivar for the integrative model estimated by genetic algorithm (GA, fields in grey) and post-GA tuning by fitting the model to the observations indicated.

	Cappricia	*Brioso*	*Moneyberg-1*	*Moneyberg-2*	*Moneymaker*	*unit*	*Tuned to observations:*
Q_{10} development	1.40	1.15	1.75	1.75	1.75	-	Time course hexoses
Q_{10} cell division	1.60	2.47	1.00	1.00	1.00	-	Cell number when ripe
Q_{10} cell expansion	3.97	4.76	1.00	1.00	1.00	-	Temperature response final fruit weight
Richards constants:							
Cell expansion:							
a_{exp}	$6.0*10^{-11}$	$5.7*10^{-11}$	$3.3*10^{-10}$	$3.3*10^{-10}$	$3.3*10^{-10}$	g FW	Final fruit weight
b_{exp}	8.23	8.15	7.70	7.70	6.53		Fruit growth curve
c_{exp}	0.46	0.47	0.65	0.65	0.55		idem
d_{exp}	2.31						Final fruit weight
Cell division:							
a_{div}	257	135	63	23	63	g FW	Final cell number
b_{div}	25						idem
c_{div}	0.07						Idem
d_{div}	1.23						Idem
L_p	0.42	0.23	0.32	0.26	0.34	g cm^{-2} MPa^{-1} h^{-1}	Final fruit weight
τ_s in σ_f	σ_f =1 - EXP(-1.69E-06*tt^2)					-	Fruit growth curve
Initial active hexose uptake c1	0.99	0.68	0.98	0.98	0.47		Hexose concentrations in time
Decrease with t of active hexose uptake	1- σ_f	1- σ_f	1- σ_f	1- σ_f	1-tt/2000	h^{-1}	Idem
Maximum mass flow M_m	0.025				0.011	µmol SUC eq. min^{-1} g^{-1} FW	Idem
Starch synthesis	5 x 2.36 (=value in Chen et al., 2021)					h^{-1}	Starch contents observed in Chen et al.
π_{F0}	0.5					MPa	
ρ	0.48#					g water cm^{-2} h^{-1} MPa^{-1}	Expected transpiration#

Note: L_p, phloem conductivity for water transport; σ_f , solute reflection coefficient between phloem vascular tissue and fruit compartment; $\pi_{F0,}$ osmotic pressure due to the solutes other than sugars in fruit; ρ, vapor permeation fruit skin; #, tuned to arrive at daily transpiration of 1% of current fruit fresh weight; tt, time x activity coefficient.

Table S5. Thermal time dependent functions of enzyme capacities derived for the tomato cultivar 'Moneymaker'.

GK $V_{max\,GK} = -3.4885*10^{-15} * (t*60)^3 + 6.2458 *10^{-10} * (t*60)^2 - 3.8249 * 10^{-5} * (t*60) + 0.8605$

FK $V_{max\,FK} = -6.7103*10^{-15} * (t*60)^3 + 1.1894*10^{-9} * (t*60)^2 - 6.9466 * 10^{-5} * (t*60) + 1.4057$

SPS $V_{max\,SPS} = 1.2652*10^{-15} * (t*60)^3 - 1.2248 *10^{-10} * (t*60)^2 + 3.4344 * 10^{-6} * (t*60) + 0.0483$

Susy $V_{max\,Susy} = 7.9541*10^{-15} * (t*60)^3 -1.1936 *10^{-9} * (t*60)^2 +4.8084 * 10^{-5} * (t*60) + 0.0113$

NI $V_{max\,NI} = \left(3.8035*10^{-16} * (t*60)^3 -7.9214 *10^{-13} * (t*60)^2 - 3.0429 * 10^{-6} * (t*60) + 0.2028\right)/2$

PFK $V_{max\,PFK} = -8.4421*10^{-16} * (t*60)^3 + 1.4446 *10^{-10} * (t*60)^2 – 8.6909 * 10^{-6} * (t*60) + 0.2490$

PFP $V_{max\,PFP} = -1.5405*10^{-14} * (t*60)^3 + 2.6240 *10^{-9} * (t*60)^2 – 1.5537 * 10^{-4} * (t*60) + 3.7471$

AI $V_{max\,AI} = \left(2.5979*10^{-14} * (t*60)^3 – 2.726 *10^{-9} * (t*60)^2 + 7.4956 * 10^{-5} * (t*60) + 0.2481\right)/2$

PGI $V_{max\,PGI} = -1.0305*10^{-14} * (t*60)^3 + 1.7290 *10^{-9} * (t*60)^2 – 9.9270 * 10^{-5} * (t*60) + 2.4434$

PGM $V_{max\,PGM} = -2.9019*10^{-14} * (t*60)^3 + 4.7246 *10^{-9} * (t*60)^2 – 2.7928 * 10^{-4} * (t*60) + 7.3772$

UGPase $V_{max\,UGPase} = -4.8047*10^{-15} * (t*60)^3 +5.7552 *10^{-10} * (t*60)^2 -3.1225 * 10^{-5} * (t*60) +3.9769$

ALD $V_{max\,ALD} = -5.1582*10^{-14} * (t*60)^3 + 9.2647 *10^{-9} * (t*60)^2 – 5.4484 * 10^{-4} * (t*60) +12.7342$

FBPase $V_{max\,FBPase} = -5.725*10^{-18} * (t*60)^3 + 1.6521 *10^{-12} * (t*60)^2 – 2.0406 * 10^{-7} * (t*60) + 0.0185$

Footnote: Temperature-modified time t = time * activity coefficient, in minutes; listed values are valid for air temperature of 21 °C, at other temperatures the V_{max} is multiplied with the activity coefficient based on a Q_{10}-enzymes of 1.4 (see Equation 16 in protocol S1 for details)

Table S6 Fruit subcellular volume fractions, nucleotide concentrations, and vacuolar ΔpH for the integrative model

Parameter	Value or equation	Reference
Volume fraction of vacuole (Vol_{vac})	0.853*(1.0-exp((-2292-60*t)/10633))	Beauvoit *et al*. (2014)
Volume fraction of cytoplasm (Vol_{cyt})	(0.933-Vol_{vac})/1.13;	
Volume fraction of plastid (Vol_{plast})	Vol_{cyt}*0.13	
ATP	$106.846*(60*t)^{-0.745}$	
UTP	ATP/3	
ADP	$(21.172/3)*(60*t)^{-0.742}$	
UDP	ADP/3	
Pi	4.7	
PPi	0.034	
Vacuolar ΔpH	$-6.3066*10^{-5}*(t/24)^{3}+6.3636*10^{-3}*(t/24)^{2}-0.1866*t/24+4.2311$	Chen *et al*. (2021)

Footnotes: Concentrations of ATP, UTP, ADP, UDP, and Pi are expressed in μmol g^{-1} FW and t is the temperature-modified time (similar to Table S5) in hour. The concentration expressed in μmol g^{-1} FW can be converted to local concentration expressed in mM by multiplying (S +W)/(Vol*Volume fraction). PPi is expressed in mM.

Supplementary references

1. Bertin N, Heuvelink E. 1993. Dry-matter production in a tomato crop: comparison of two simulation models. Journal of Horticultural Science **68**, 995-1011.

2. Heuvelink E. 1995. Dry matter production in a tomato crop: measurements and simulation. Annals of Botany (Lond.) **75**, 369–379.

3. Okello RCO, Heuvelink E, de Visser PHB, Lammers M, de Maagd RA, Marcelis LFM, Struik PC. 2014. A multi-level analysis of fruit growth of two tomato cultivars in response to fruit temperature. Physiologia Plantarum 153, 403–418.

4. Adams S, Cockshull K, Cave C. 2001. Effect of temperature on the growth and development of tomato fruits. Annals of Botany **88**, 869–877.

5. Fanwoua J, de Visser PHB, Heuvelink E, Angenent G, Yin X, Marcelis LFM, Struik PC. 2012. Response of cell division and cell expansion to local fruit heating in tomato fruit. Journal of the American Society for Horticultural Science **137**, 294-301.

6. Ruan Y-L, Patrick JW. 1995. The cellular pathway of postphloem sugar transport in developing tomato fruit. Planta **196**, 434-444.

7. Cosgrove DJ. 1985. Cell wall yield properties of growing tissue evaluation by in vivo stress relaxation. Plant Physiology **78**, 347-356.

8. Grange R. 1995. Water relations and growth of tomato fruit pericarp tissue. Plant, Cell & Environment **18**, 1311-1318.

9. Fishman S, Génard M. 1998. A biophysical model of fruit growth: simulation of seasonal and diurnal dynamics of mass. Plant, Cell & Environment **21**, 739-752.

10. De Swaef T, Steppe K. 2010. Linking stem diameter variations to sap flow, turgor and water potential in tomato. Functional Plant Biology **37**,429–438.

11. Liu HF, Génard M, Guichard S, Bertin N. 2007. Model-assisted analysis of tomato fruit growth in relation to carbon and water fluxes. Journal of Experimental Botany **58**, 3567-3580.

12. Chen J, Beauvoit B, Génard M, Colombié S, Moing A, Vercambre G, Gomes E, Gibon Y, Dai Z. 2021. Modelling predicts tomatoes can be bigger and sweeter if biophysical factors and transmembrane transports are fine-tuned during fruit development. New Phytologist **230**, 1489-1502

13. Milner I, Ho L, Hall J. 1995. Properties of proton and sugar transport at the tonoplast of tomato (Lycopersicon esculentum) fruit. Physiologia Plantarum **94**, 399-410.

14. Beauvoit BP, *et al*. 2014. Model-assisted analysis of sugar metabolism throughout tomato fruit development reveals enzyme and carrier properties in relation to vacuole expansion. The Plant Cell 26, 3224-3242.

Supplementary protocol S1. Model on sugar and water flows at fruit level.

Water inflows from phloem and xylem:

$U_p = A_p L_p [P_p - P_f - \sigma_p (\pi_p - \pi_f)]$ (1)

$U_x = A_x L_x [P_x - P_f - \sigma_x (\pi_x - \pi_f)] = A_x L_x [P_x - P_f + \pi_f]$, since $\sigma_x = 1$ and $\pi_x = 0$ (2)

Here $A_p = A_x = axp * A_f$, with $A_f = 4 \pi (0.5 Ø_f)^2$, and $Ø_f = 2 (V_f 3/(4\pi))^{1/3}$, with $V_f = FFW (1 - 0.5 FDW)$ assuming 0.5 part of fruit dry weight consist of (an)organic compounds dissolved in water and the other 0.5 part is solid with a specific density of approximately 1 kg L^{-1}.

If $P_f \geq Y$:

Fruit volume growth per hourly time step: $dV_f/dt = (U_x + U_p - T_f - V_n)/D_w + (U_a - R_f)/D_s$ (3)

Also:

$dV_f/dt = V \Phi (P_f - Y)$ (4)

Combining Equations 3 and 4, and using eqs (1), (2) and $\Delta\pi=(\pi_p - \pi_f)$ to rewrite U_p and U_x, we obtain:

$P_f = [A_x L_x (P_x + \pi_f) + A_p L_p (P_p - \sigma_p \Delta\pi) - T_f - V_n D_w V_f \Phi Y] / (A_x L_x + A_p L_p + D_w V_f \Phi)$ (5)

Where fruit transpiration ($m^3 h^{-1}$) is:

$T_f = A_f c_T P^* 10^{-6} (H_f - H_a)$ (6)

The meaning of the variables in eqs 1 to 6 is as follows:

A = surface area of transport route or fruit skin (m^2)

axp = proportionality constant between area of vascular tissue and fruit skin area (-)

L = hydraulic conductivity ($m^3 m^{-2}$)

σ = solute reflection coefficient of membranes between fruit symplast and phloem (σ_p) or xylem (σ_x) (-)

P = hydrostatic pressure (MPa)

Y = threshold hydrostatic pressure above which irreversible fruit expansion occurs (MPa)

π = osmotic pressure (MPa)

V_n = volume of new formed cells n per time step (m^3 per fruit)

V = current fruit volume (m^3)

D = specific density of water (D_w) and sugar (D_s) (kg dm^{-3})

Φ = coefficient for cell wall extensibility (-)

c_T = transpiration coefficient (g m^{-2} hr^{-1} MPa^{-1})

P^* = saturated vapor pressure (Pascal) at the ambient temperature (Matlab function Td2svp(T), with T in °C, see Supplementary protocol S2 and Psychometry Handbooks, MartinD@eeng.dcu.ie)

H = relative humidity of the air-filled space in the fruit (H_f) and of the ambient atmosphere (H_a) (-)

Subscripts refer to phloem (p), xylem (x) and fruit (f)

Sugar uptake:

Sucrose (SUC) arrives solely through the phloem in fruit tissue, via mass flow or diffuse flow. The mass flow has to pass a membrane with an increasing reflection for solutes, σ_p, which is a function of time t (hours) multiplied with an activity coefficient (Eq. 16) with a Q_{10} specific for σ_p development to tt (thermal hours or °Ch, see also Supplementary Table S4b) - and a coefficient τ_s affecting the shape of the curve:

$\sigma_{p} = (1 - \exp(-\tau_s * tt^2)$ (-) (7)

Thus, the equation for mass flow (Equation 8) combines the above mentioned water flux U_p, the increasing reflection of solute σ_p, and an upper threshold for massflow flux M_m (see its fitted value in Supplementary Table S4b) (backflow of water never occurred and is thus not incorporated):

$F_{suc,\ massflow} = \max (U_p (1 - \sigma_p) [suc_p], M_m)$ (mol m^{-2}) (8)

The diffusion of sucrose over a concentration gradient, with a symplasm permeability p_{suc} that decreases over time, is described in Equation 9:

$F_{suc,\ diffusion} = A_p\, p_{suc} ([suc_p] - [suc_s])$ (mol m^{-2}) (9)

Active sugar uptake is achieved by a membrane carrier to transport hexose (fructose, glucose) over a membrane from apoplast to symplast. This modelled process is temperature-sensitive and thus multiplied with a Q_{10} of 1.4 being our default for enzymatic processes. The transport capacity of the membrane for hexose has an initial maximum c1 (see Supplementary Table S4b) and is assumed to decrease in proportion to the increase of the solute reflection coefficient of phloem σ_p (as used for mass flow in Eq.7) and is described in Equation 10:

Active hexose uptake $U_a = c1 * Q_{10} * (1 - \sigma_p)$ (mol sucrose equivalents m^{-2}) (10)

For cv. Moneymaker we had to construct a different curve shape in order to fit to the observed course of hexose concentrations, and this active uptake is described in Equation 11, using a different value for c1 and a slope parameter c2 for linear decrease of the uptake with thermal time:

Active hexose uptake $U_a = c1 * Q_{10} * (1 - tt/c2)$ (mol sucrose equivalents m^{-2}) (11)

The resulting gross dry matter growth following sugar uptake is:

$dS_{gross}/dt = F_{suc,\ massflow} + F_{suc,\ diffusion} + U_a$ (in mol sucrose equivalents m^{-2}) (12)

Net growth is the resultant of gross growth minus respiration from maintenance R_m and growth R_g:

$dS_{net}/dt = dS_{gross}/dt - R_f - R_g$, where $R_m = \{dS_{gross}/dt) * q_{m293} * Q_{10})$ and $R_g = ((dS_{gross}/dt) - R_m\} * (q_g - 1)$(13)

where Q_{m293} is the maintenance coefficient at 293 °K and q_g is the assimilate requirement coefficient (see Supplementary Table S4a).

Parameter estimates:

The values for proportion between vascular area and fruit area (axp), phloem and xylem hydraulic conductivity (L_p and L_x), permeation coefficient of skin for water (ρ) and the membrane permeability for sugar diffusion (p_{suc}) are listed in Supplementary Tables S4a and S4b, while the reflection coefficient for solutes exchanged through xylem (σ_x) is =1.

Model on cell dynamics and potential fruit growth:

$$\text{Thermal time(x)} = \sum_{n=1}^{24} \left(\frac{T_{air}(x) - T_{base}}{24} \right) \qquad \text{(d°C)} \qquad (14)$$

with $T_{air}(x)$ = air temperature in hour x of growth; T_{base} = base temperature (see also Table 3);

DVS(x) = Thermal time(x)/TsumRipe, where the fruit is ripe at DVS (development stage) =1; (15)

Activity coefficient = $Q_{10}^{(Tair-21)/10}$, equals the Arrhenius function (-) (16)

dCellsize/dt = Activity $\text{coefficient}_{expansion}$ * $R_{cellexpansion}$(DVS) (g h^{-1}) (17)

dCellnumbers/dt = Activity $\text{coefficient}_{division}$ * $R_{celldivision}$(DVS) (h^{-1}) (18)

with $$R(DVS) = \frac{a * b * (1 + e^{-b*(DVS-c)})^{\frac{1}{1-d}}}{(d-1) * (1 + e^{b*(DVS-c)})}$$ (=1st order derivative of 'Richards' equation) (19)

Potential fresh weight of fruit (FFW) = Cellnumbers * Cellsize (g) (20)

dFFW/dt = (dCellsize/dt * Cellnumbers) + (Cellsize * dCellnumbers/dt) (g h^{-1}) (21)

This potential weight increase relative to the current fruit weight reflects the potential faction the cell walls can stretch in volume, equaling Φ:

Φ = f * (dFFW/dt)/FFW (-) (22)

and this value enters in Equations 4 and 5 (above).

Supplementary protocol S2. MATLAB code of the integrative model on sugar kinetics and fruit growth.

```
% FRUIT MODEL with time MINUTE

close all
clear all
y0 = [ 0.9 %SUC in umol g-1 FW
0.0425 % GLCcyt
0.0425 %FRUcyt
0.05 % GLC6P
0.001 % GLC6P_plast
0.01 %FRU6P
0.01 %UDPGLC
0.01 %S6P
3.% SUCvac
40.; % GLCvac
22.; % FRUvac
0. % FBP
0.1 % G1P
0 % SinkAll
80 % Cellwall
12 % Starch
0.95 % FFW
140  % Fat & protein
2.044*1E+06 % CellNr
7.65*1E-08 % Cellsize
0 % dFFW_FG_pot
0 % transpiration/fr
0 % inflow S
0 % SinkAll
0 % CH2O rest
];

gMOLSUC = 342.3; gMOLHEX=180.2;

%% MODEL
% Time step in minutes, so 24*60 (Tc) per day

% Choose temperature treatment:
%Tair=21; Tc = 24*60; daystart=4; days = 55; steps=7;
Tair=25; Tc = 24*60; daystart=4; days = 46; steps=6;

tstart = daystart*Tc; tfinal = days*Tc;
tx=tstart:1:tfinal;
th(1)=2.814;th(2)=0.9799 ;th(3)=3.9670;th(4)= 8.2316;th(5)= 0.4578; % GA result

[T,y]=ode23s(@(t,x) odesSUGARS(t,x,th,Tair),tx,y0); %
```

```
% OUTPUT
imax = floor(tfinal - tstart);

Tpf_min(1)=0;
for i = 2 : imax+1
Tpf_min(i)=y(i,22)-y(i-1,22);
end

Fruitsize=y(imax,17)
CumTranspiration=y(end,22)

%% sum of sugar concentrations at harvest, all in umol g-1 FW
Sugars = ((y(end,1) +y(end,9))*gMOLSUC/gMOLHEX ... % SUC: translate from suc to hex equiv
         +y(end,2)+y(end,10) ... %GLCall
         +y(end,3)+y(end,11) ... %FRUall
         +y(end,4)+y(end,5) ...  %G6Pall
         +y(end,6) ...        %F6Pall
         +y(end,7) ...        %UDPGLC
   +y(end,13) ...        %G1P
    +y(end,8) *gMOLSUC/gMOLHEX ... %S6P: also translate from suc to hex equiv
   +y(end,12) ) * gMOLHEX/1000000  %FBP  % x umol X (mol weight in g / from umol to mol)

Solids= (y(end,15) + y(end,16)+y(end,18))* gMOLHEX/1000000.
DM = Sugars+Solids %+Solids2(i);

% observed data to plot
DayOut25C = [11;18;25;32;39;46]; %at 25C, last date (53) omitted
DayOut21C = [11;18;25;32;39;46;53];
MinOut25C = DayOut25C.*Tc;
MinOut21C = DayOut21C.*Tc;
FWobs21C = [3;16;31;45;58;67;75];  %Control
FWobs25C = [2;26;44;58;63;66];    %Heated
DayHEXobs=   [19;25;30;40;50].*Tc; %
HEXcell21C = [173;159;148;127;129];%Control
HEXcell25C = [173;167;155;130;129];%Heated

% simulated hexose (glucose + fructose) tissue concentrations (umol g-1 FW)
HEXsim=y(:,2)+y(:,3)+y(:,10)+y(:,11);

% output values to be used in GA for parameter fitting
tstep = 7*Tc; tfinal2 = steps*7*Tc; Fmodel=zeros(steps,1);
Ymodel=zeros(steps,1); TimeF=zeros(steps,1);I=0;

for i = tstep : tstep: tfinal2  %start i at sim start day (4) + one step (7) = 11*Tc
  I= I+1;
  HEXmodel(I)=y(i,2)+y(i,3)+y(i,10)+y(i,11);
  FFWmodel(I)=y(i,17);
  TimeF(I)=i+4*Tc;
```

```
end

%xlswrite(['OutFFW_C.xlsx'], y(:,17));

figure('Position',[100 70 1200 700]) %[left, bottom, width, height]

subplot(2,4,1) % subplot(nr of grid rows, nr of grid columns, subplot position)
p1 = plot(tx,y(:,15),'-r',tx,y(:,16),'-b',tx,y(:,18),'*g',DayHEXobs,HEXcell21C, ...
    '*b',DayHEXobs,HEXcell25C,'*r',tx,HEXsim,'--r');
hold on
ylabel('Cum hex+suc,cellwall&starch in umol/g FW')
xlabel('  hour  ')
legend('cellwall', 'starch','fat','obs21C','obs25C')

subplot(2,4,2)
p1 = plot(tx,y(:,19),'-r',66208,2.25*1E+07,'*g');
hold on
legend('Cell number','Observed')

subplot(2,4,3)
p1 = plot(tx,y(:,20),'-b',1270*60,3.4*1E-06,'*b',1032*60,2.9*1E-06,'*r');
hold on
legend('Pot cell size','Control','Heated')

subplot(2,4,4)
p2 = plot(MinOut25C, FWobs25C,'*b',tx,y(:,17),'-r',tx,y(:,21),'-g',MinOut21C,FWobs21C,'*r');
hold on
ylabel('FFW in g')
legend('obs25C','act','pot','obs21C')

subplot(2,4,5)
p1 = plot(tx,y(:,23),'-r',tx,y(:,24),'-b',tx,y(:,25),'-g');
hold on
ylabel('uptake and sink in umol SUC g-1 FW min-1')
legend('uptake', 'sink', 'rest kinetics')

subplot(2,4,6)
p1 = plot(tx,y(:,22),'-b');
hold on
ylabel('cum transpiration in g/g FW')
legend('Tpf')

subplot(2,4,7)
p1 = plot(tx,Tpf_min,'-r');
hold on
ylabel('Transpiration rate (g/min)')
```

```
%%%%%%%%%%%%%%%%%%%%%%%%%%%%%%%%%%%%%%%%%%%%%%%%%
function dy = odesSUGARS(tt,y,thx,Tair)
% tt in minutes

% Fitted parameters thx: 1:ChangeL, 2:ActFlS, 3:Q10-expansion, 4:RichB_, 5:RichC_
% Fully functioning ODE solver, output variables in y-vector.

% Load Parameter values:
par    = Parameters1;
param2 = Parameters2;

Source_effect_div=1;
Source_effect_Cp=1;

  diff=sin(deg2rad((tt+720)*0.25));
  Tair = Tair+diff;
  Cp_t=Source_effect_Cp*(0.08 + 0.038*diff);
  WatPot =-0.3 - 0.3*diff;
  Tair_=Tair;
Q10     = par.Q10;
Arrh=Q10^((Tair_ -21)/10);

if isstruct(param2) == 1
  param2 = struct2cell(param2(:));
end
%------------------- parameters from Parameters2.m ----------
  [gMOLSUC,gMOLHEX,KmGLCcyt_HK_GLC, KmATP_HK_GLC, KmFRUcyt_HK_GLC, ...
 KiGLC6P_HK_GLC, KiFRU6P_HK_GLC, KmATPfrka, KmFRUfrka, KeqSPS, KiS6P_SPS, ...
 KmFRU6P_SPS, KiPiSPS, KiUDPGLC_SPS, KmUDPGLC_SPS, rVfVrSPS, KmUDP_SPS, ...
 KmS6P_SPS, KiFRU6P_SPS, Pi,KmS6P_SPase, VmSPase,KiFRUssc, KmSUCssc, ...
 KiUDPssc, KmUDPssc, KeqSS, KmUDPGLCssc, KmFRUssc,KiSUCssc,rVfVrSusy, ...
 KiGLUcytNI,KmSUC_NI,KiFRUcytNI,FRU6Phalf, ATPhalf, hpfk,ModATPhalf, sigma, ...
 KeqPfP, KmFRU6Ppfp, KmFBPpfp, KmPPipfp, KmPipfp, Km10Suc, KiGLCvacAI, ...
 KmSUCvacAI, KiFRUvacAI,Km16Glc,Km17G6P, Km17F6P,rVfVrPGI,Km18G6P, ...
 Km18G1P,rVfVrPGM, Km19UTP, Km19G1P, Km19PPi, Km19UDPGLC,rVfVrUGPase, ...
 KmFBPALD, Km23G6P, Km23Piplast, Km23G6Pplast, Km23Pi,r_Pi, PPi, KmFBP] = param2{:};
%------------------------------------------------------------------------

Tbase = 3;
Q10_development = 1.4;

t=tt*Arrh;
Vvac= 0.853*(1.0-exp((-2292-t)/10633));
Vcyt = (0.933-Vvac)/1.13;
Vplast = Vcyt*0.13;
  rho1=((.0775* t)/1440+13);
  rho2=((.0775* t)/1440+12);
  rho=rho1/rho2; % in g FW mL-1 tissue
```

```
uMol_to_mM_cyt = rho/Vcyt;
uMol_to_mM_vac = rho/Vvac;
uMol_to_mM_plast = rho/Vplast;

%  convert y-value (umol equiv g-1 FW) to mM in cytoplasm, vacuole or plastid
SUC = y(1)*uMol_to_mM_cyt;
GLCcyt = y(2)*uMol_to_mM_cyt;
FRUcyt = y(3)*uMol_to_mM_cyt;
GLC6P=y(4)*uMol_to_mM_cyt;
GLC6P_plast=y(5)*uMol_to_mM_plast;
FRU6P=y(6)*uMol_to_mM_cyt;
UDPGLC=y(7)*uMol_to_mM_cyt;
S6P=y(8)*uMol_to_mM_cyt;
SUCvac=y(9)*uMol_to_mM_vac;
GLCvac=y(10)*uMol_to_mM_vac;
FRUvac=y(11)*uMol_to_mM_vac;
FBP=y(12)*uMol_to_mM_cyt;
G1P=y(13)*uMol_to_mM_cyt;
%below is used as umol g-1 FW:
Cellwall=y(15);
Starch=y(16);
% fruit fres weight:
FFW_old=y(17);
% fat & protein concentration has a forced, linear time pattern:
Fat=y(18);

%all Vmax are in unit umol min-1 g-1 FW (so cell scale)
VmGK=Arrh*(-3.4885*1E-15*t^3 + 6.2458*1E-010*t^2 - 3.8249*1E-05*t + 0.8605);
VmFK=Arrh*(-6.7103*1E-15*t^3 + 1.1894*1E-09*t^2 - 6.9466*1E-05*t + 1.4057);
VmSPS= Arrh*(1.2652*1E-15*t^3 - 1.2248*1E-10*t^2 + 3.4344*1E-06*t + 0.0483);
VmSuSy= max(0,Arrh*(7.9541*1E-15*t^3-1.1936*1E-9*t^2+4.8084*1E-05*t+0.0113));
VmPFK = Arrh* (-8.4421*1E-16*t^3+1.4446*1E-10*t^2-8.6909*1E-06*t+0.2490);
VmNI= Arrh *(3.8035*1E-16*t^3-7.9214*1E-13*t^2-3.0429*1E-06*t+0.2028)/2;
VmAI= Arrh*(2.5979*1E-14*t^3-2.726*1E-09*t^2+7.4956*1E-05*t+0.2481)/2;
VmPFP= Arrh*(-1.5405*1E-14*t^3+2.6240*1E-09*t^2-1.5537*1E-04*t+3.7471);
VmPGI = Arrh*(-1.0305*1E-14*t^3+1.7290*1E-09*t^2-9.9270*1E-05*t+2.4434);
VmPGM = Arrh*(-2.9019*1E-14*t^3+4.7246*1E-09*t^2-2.7928*1E-04*t+7.3772);
VmUGPase=Arrh*(-4.8047*1E-15*t^3+5.7552*1E-10*t^2-3.1225*1E-05*t+3.9769);
VmALD= Arrh*(-5.1582*1E-14*t^3+9.2647*1E-09*t^2-5.4484*1E-04*t+12.7342);
VmGPT=Arrh*0.206;
VmFBPase=Arrh*(-5.725*1E-18*t^3+1.6521*1E-12*t^2-2.0406*1E-07*t+0.0185);
% ORIGINAL CODE CHANGED TO MINUTES (/60):
Vm10=Arrh*0.0326*exp(exp(0.008574*(t/60-1209.26))+exp(-0.001714*t/60));
Vm16= Arrh*0.0344*exp(-0.001874 * t/60);

ATPg=Arrh*106.846*t^(-0.745);
UDPg=Arrh*(21.172/3)*t^(-0.742);
% now convert to mM to be used in MM equations:
```

```
ATP=ATPg*uMol_to_mM_cyt; %
UTP=ATP/3.;
UDP=UDPg*uMol_to_mM_cyt; %
deltapH= (-6.3066*1E-05*(t/1440)^3 +6.3636*1E-03*(t/1440)^2 -0.1866*(t/1440)+4.2311);

% All kinetic fluxes  in umol min-1 g-1 FW, their concentrations in mM
% N.B. transport rates (carriers V10,V12-14,V23) and storage (V12-14,V21,V22)
% with conc in umol g-1 FW, their flux output ALSO in umol HEX equivalent min-1 g-1 FW

V2=VmGK*(GLCcyt/KmGLCcyt_HK_GLC)*((ATP/KmATP_HK_GLC)/((1.0+ATP ...
 /KmATP_HK_GLC)*(1.0+GLCcyt/KmGLCcyt_HK_GLC+FRUcyt/KmFRUcyt_HK_GLC ...
 +GLC6P/KiGLC6P_HK_GLC+FRU6P/KiFRU6P_HK_GLC))); % GK
V3=VmFK*FRUcyt*(ATP/(KmATPfrka*FRUcyt+KmFRUfrka*ATP+FRUcyt*ATP)); % FK
V4=VmSPS*(FRU6P*UDPGLC-S6P*UDP/KeqSPS)/(FRU6P*UDPGLC*(1.0+S6P ...
 /KiS6P_SPS)+KmFRU6P_SPS*(1.0+Pi/KiPiSPS)*(UDPGLC+KiUDPGLC_SPS) ...
 +KmUDPGLC_SPS*FRU6P+(rVfVrSPS/KeqSPS)*(S6P*KmUDP_SPS*(1.0+ ...
 UDPGLC/KiUDPGLC_SPS)+UDP*(KmS6P_SPS*(1.0+KmUDPGLC_SPS*FRU6P/ ...
 (KiUDPGLC_SPS*KmFRU6P_SPS*(1.0+Pi/KiPiSPS)))+S6P*(1.0+FRU6P/ ...
 KiFRU6P_SPS)))); %SPS
V5=Arrh*VmSPase*S6P/(KmS6P_SPase+S6P); % SPase
V6=(1.* (-VmSuSy))*(SUC*UDP-FRUcyt*UDPGLC/KeqSS)/(SUC*UDP*(1.0+FRUcyt/ ...
 KiFRUssc)+KmSUCssc*(UDP+KiUDPssc)+KmUDPssc*SUC+(rVfVrSusy/KeqSS)*(FRUcyt* ...
 KmUDPGLCssc*(1.0+UDP/KiUDPssc)+UDPGLC*(KmFRUssc*(1.0+KmUDPssc* ...
 (SUC/(KiUDPssc*KmSUCssc)))+FRUcyt*(1.0+SUC/KiSUCssc)) )); % SuSy
V7=(VmNI/(1.0+GLCcyt/KiGLUcytNI)) * (SUC/(KmSUC_NI*(1.0+FRUcyt/KiFRUcytNI)+SUC));
V8counter=VmPFK*(FRU6P/FRU6Phalf)^hpfk*(ATP/ATPhalf)^hpfk;
V8denom=(1.0+(ATP/ModATPhalf)^hpfk)/(1.0+sigma^(4.0*hpfk)*(ATP/ModATPhalf)^hpfk)+ ...
   ((FRU6P/FRU6Phalf)^hpfk+(ATP/ATPhalf)^hpfk) * (1.0+sigma^(2.0*hpfk) *(ATP/ATPhalf) ...
   ^hpfk )/(1.0+sigma^(4.0*hpfk) *(ATP/ATPhalf)^hpfk )+(FRU6P/FRU6Phalf)^hpfk ...
   *(ATP/ATPhalf)^hpfk;
V8=V8counter/max(0.01,V8denom); % avoid zero division
V9=(VmPFP/(KmFRU6Ppfp*KmPPipfp))*(FRU6P*PPi-FBP*Pi/KeqPfP)/ ...
     ((1+FRU6P/KmFRU6Ppfp+FBP/KmFBPpfp)*(1+PPi/KmPPipfp+Pi/KmPipfp));
Km10Sucvac = Km10Suc*10^deltapH;
V10=(Vm10*(SUC/Km10Suc-
SUCvac/Km10Sucvac))/(1.0+SUC/Km10Suc+SUCvac/Km10Sucvac);
V11=((VmAI/(1.0+GLCvac/KiGLCvacAI))*SUCvac/(KmSUCvacAI*(1.0+FRUvac/ ...
      KiFRUvacAI)+SUCvac));
Km16Glcvac = Km16Glc*10^deltapH;
V15=(Vm16*(GLCcyt/(Km16Glc+FRUcyt)-GLCvac/(Km16Glcvac+FRUvac)))/ ...
       (1.0+GLCcyt/(Km16Glc+FRUcyt)+GLCvac/(Km16Glcvac+FRUvac));
V16=(Vm16*(FRUcyt/(Km16Glc+GLCcyt)-FRUvac/(Km16Glcvac+GLCvac)))/ ...
      (1.0+FRUcyt/(Km16Glc+GLCcyt)+FRUvac/(Km16Glcvac+GLCvac));
V17=VmPGI*(GLC6P/Km17G6P-FRU6P/(rVfVrPGI*Km17F6P)) / ...
      (1.0+GLC6P/Km17G6P+FRU6P/Km17F6P);
V18=VmPGM*(GLC6P/Km18G6P-G1P/(rVfVrPGM*Km18G1P)) / ...
       (1.0+GLC6P/Km18G6P+G1P/Km18G1P);
V19=(VmUGPase*(UTP*G1P/(Km19UTP*Km19G1P)-UDPGLC*PPi/(rVfVrUGPase* ...
```

```
    Km19UDPGLC*Km19PPi)))/((1.0+UDPGLC/Km19UDPGLC+UTP/ ...
    Km19UTP)*(1.0+PPi/Km19PPi+G1P/Km19G1P));
V20_2=(VmALD*FBP/(KmFBPALD+FBP));
Piplast = r_Pi*Pi;
V23=((VmGPT/(Km23G6P*Km23Piplast))* (GLC6P*Piplast - GLC6P_plast*Pi)) / ...
  ((1+GLC6P/Km23G6P+GLC6P_plast/Km23G6Pplast)*(1+Piplast/Km23Piplast+Pi/Km23Pi));
V24=(VmFBPase*FBP/(KmFBP+FBP));


% All in- or output (UDPGLC, G6P_plast) are concentrations at FW scale (concentrations used
% for the enzyme kinetics (mM) were converted from umol g-1 FW to mM, by dividing with
% uMol_to_mM):
t1 = t/60; %t1 is in hour, t in minutes, all thermal
CorrSynStarch=5;
K_synstarch = CorrSynStarch*2.386/(1+exp((t1-768.72)/166.32));
V25 = (1/uMol_to_mM_plast) * GLC6P_plast * K_synstarch;
V25=V25/60; %convert hourly flux to minute
V25d = Arrh*Starch * 0.0187;
V25d=V25d/60;

k_synCellwall = 110.47/(1+exp((t1-680.64)/496.8));
V26 =  Arrh*(1/uMol_to_mM_cyt) * UDPGLC * k_synCellwall;
V26=V26/60;
V26d = 0.00306 * Cellwall;
V26d=V26d/60;

SinkSUC = max(0.,V10+(-V5+V6+V7)); % total sugar sink (umol g-1 FW min-1)

%% home-made module on cell division and expansion
TsumRipe=992;
% CHANGED, ThermalTime per minute, so /60:
ThermalTime=tt*(Tair-Tbase)/(24*60);
DVS = ThermalTime/TsumRipe;
DVS_DPA0=-0.1;
DVSnew = min(1.0,DVS+DVS_DPA0);
% rate of cell expansion (x 60 to get growth per hour):
RichA_=0.6*1E-010;
RichB_ = thx(4);
RichC_= thx(5);
RichD_ =2.31;
CorrRate_ = thx(3)^((Tair_ -21)/10);

Cellnr=y(19);
Cellsize=y(20);
Increase_cell2 = ((RichA_*RichB_*(1+exp(-RichB_*(DVSnew-RichC_)))^(1/(1-RichD_))) ...
                    /((RichD_-1)*(exp(RichB_*(DVSnew-RichC_))+1)))*CorrRate_;
fi_cell = Increase_cell2/Cellsize; % potential cell extension
```

```
% rate of cell division:
DVScorr_div = 1;
RichA=135.*1.9 ;
RichB=25. ;
RichC=0.07 ; RichD =1.23 ;
CorrRate = 1.6^((Tair_ -21)/10);
Increase_cellnr2=((RichA*RichB*(1+exp(-RichB*(DVSnew-RichC)))^(1/(1-RichD))) ...
                    /((RichD-1)*(exp(RichB*(DVSnew-RichC))+1)))*CorrRate*DVScorr_div;

FFW_FG_pot = (Cellnr + Increase_cellnr2) * (Cellsize + Increase_cell2);
dRich = FFW_FG_pot - Cellnr * Cellsize;
dFFW_FG_pot_div = Increase_cellnr2 * 7.65*1E-08; % new cells have initial size
dFFW_FG_pot = max(0, dRich);

%% Biophysical model
% set initial conditions
Temp   = Tair + 273.15; % temperature (K)
RH     = 75.0;

% TempEffect for active enzymic processes (uptake, maintenance):
TempEffect =Q10^((Temp-293.15-1.0)/10.0);
i=tt*Q10_development^((Tair_-21)/10.0);% thermal time approach, i in 0Cm

DissolvedSugarsCyt = y(1)+y(2)+y(3)+y(6)+y(7)+y(8)+y(12)+y(13);
FractionFatProt_inOSMf=0.7; %0.7 estimate
SolubleFatProtein = FractionFatProt_inOSMf*max(0,Fat); % dissolved fat/proteins in cytoplasm
SolidFatProtein = max(0, Fat - SolubleFatProtein); % rest is built in fat, acids, proteins
SugarsOrganelles = y(5)+y(9)+y(10)+y(11)+Fat;
SugarsVac= y(9)+y(10)+y(11);

SUC_g = SUC*gMOLSUC/1E+06; %in g g-1 FW
% DM in g g-1 FW; 0.02=proteins,acids,DNA,fibers:
DM   = 0.02+(y(15)+y(16)+DissolvedSugarsCyt+SugarsOrganelles)*gMOLHEX/1E+06;

%parameter values from Parameters1.m
acnst = par.acnst;
qg     = par.qg;
rwtr   = par.rwtr;
R      = par.R;
Hf     = par.Hf;
Y      = par.Y;
Y0     = par.Y0;
paa    = par.paa;
Mm     = par.Mm; % Mm 0.025 (all cultivars) but Moneymaker 0.011
Km     = par.Km; % (Milnder, 1995)
con    = par.con;
```

```
%Time-related parameters changed to minute
qm20    = par.qm20/60;
Lp      = par.Lp/60 ; % hydraulic conductivity phloem (g cm-2 MPa-1, now per min)
Lx      = par.Lx/60 ; % hydraulic conductivity xylem; IDEM
PermS   = par.PermS/60; % permeation tomato pericarp phloem membrane, per min.

% set states in the Fishman&Genard model:
Cp      = Cp_t; % now with diurnal variation
FDW = DM*FFW_old;
% 50% of dry matter is dissolved in water thus not adding volume; rest of mass in g = mL:
Fvol    = FFW_old-0.5*FDW;
Fdia    = 2*(Fvol*3/(4*pi))^(1/3);          %cm
sigma = 1-exp(-0.00000169*(i/60)^2); % S-shape, ending at 0.95 at harvest
molSUCp    = Cp/gMOLSUC * 1E+06;
OSMp      = 0.7+paa * molSUCp *R*Temp*1E-06;
Af        = 4*pi*(Fdia/2)^2;                 % cm2 fr-1
Aw        = Af*acnst;                        % cm2 fr-1; conductory vessels (xylem,phloem)area
Ha        = RH/100;                          % relative humidity in the ambient atmosphere
PstarNew   = Td2svp(Temp-273.15);            % "svp" in Pascal, see below for Matlab model by
                                             % MartinD@eeng.dcu.ie
OSMx      = 0;                               %no solutes in xylem, so osm.pot. = 0
PTLx      = WatPot - OSMx;                   % Turgor Xylem (MPa)
Tpf       = Af*con*(PstarNew/1000000)*(Hf-Ha);
OSMf = 0.5 + paa * (SugarsVac+SolubleFatProtein) * 8.3144  * Temp * 1E-06;
TURGp      = PTLx+OSMp;                      % MPa
Tune_fi_cell = 1.8; %scaling factor
% fi_cell (extensibility) derived from growth in Richards equation (in minutes):
fi= Tune_fi_cell*fi_cell;
eps = fi*FFW_old;
ChangeL = thx(1);
Lx = ChangeL * Lx;
Lp = Lp * ChangeL;

%Solving Fishman & Genard (1998) equations: time step is minute:
BIDA       = Aw*Lx*PTLx+Aw*Lx*OSMf+Aw*Lp*TURGp- ...
             Aw*Lp*sigma*(OSMp-OSMf); % counter (part 1) of Eq. 15
BIDB       = eps*Y-Tpf-dFFW_FG_pot_div*(1-DM); % counter (part 2) of Eq. 15
TURGf      = (BIDA + BIDB)/(Aw*Lx+Aw*Lp+eps);
TURGf      = max(Y0,TURGf);

difPTLx   = PTLx-TURGf-1.0*(OSMx-OSMf);
Uwx       = max(0,Aw*Lx*difPTLx);
difPTLp   = TURGp-TURGf- sigma*(OSMp- OSMf);
Uwp       = max(0,Aw*Lp*difPTLp);

%sugar uptake - passive, mass flow, and active, in g SUC min-1 fr-1.
pasflS = max(0,Aw*PermS*(Cp-SUC_g));
masflS = (1.0-sigma)*Uwp*Cp;
```

```
% and then in umol SUC min-1 g-1 fruit fresh weight:
pasflS_umol = (pasflS*1E+06/gMOLSUC)/FFW_old;
masflS_umol=min(Mm,(masflS*1E+06/gMOLSUC)/FFW_old);

% Active hexose uptake (IN SUCROSE EQUIVALENTS)

ActFlS=thx(2);% tuned to observations
% METHOD MONEYMAKER:
%activS_umol=0.0167*TempEffect*0.47*(1-i/120000);

% METHOD steep linear decline
%activS_umol=max(0,TempEffect*0.0167*ActFlS*(1-i/63250));

% DEFAULT METHOD: active uptake = f(sigma)
activS_umol=TempEffect*0.0167*ActFlS*(1-sigma);

% METHOD Chen:
%     tau=369.53; tStar= 273.25; % both in unit hour
%     CorrActFlChen=0.7;% tuned to observations
%     Vm = CorrActFlChen*0.01584; % in g sucr equiv g DW h-1
%     % TIMESTEP FROM i TO HOURS
%     VmaxUP=Vm/(1+exp((i/60 - tStar)/tau));
%     % HOURLY OUTPUT VALUE CONVERTED TO MINUTE (1/60 = 0.0167):
%     ActUptake_gFW = 0.0167*(VmaxUP*Cp/(Km+Cp))*FDW/FFW_old;
%     activS_umol= ActUptake_gFW*1E+06/gMOLSUC;

activS=activS_umol*FFW_old*gMOLSUC/(1E+06);

%% respiration costs:
qm        = qm20*TempEffect; % maintenance respiration in g CH2O per g DW min-1
Rf_maint        = qm*FDW;    % maintenance respiration in g CH2O per fruit min-1

%for growth, in umol SUC per g FW per minute:
V1 = masflS + pasflS + activS;
Us_gross = V1 - Rf_maint ; % in g CH2O min-1 fruit-1

%Actual dry matter and water increment (g), per fruit per time step:
Ds        = Us_gross * 1./(1+qg) ; % actual, net growth
Dw        =  max(0,Uwx+Uwp+rwtr*Rf_maint -Tpf); % Water uptake
dFFW_FG_V1     = Dw + Ds;

%Actual fluxes for ode-solver: % all S flows, umol SUC g-1 FW min-1;
V0_umolSUC=masflS_umol+pasflS_umol;
actflS_umolSUC=activS_umol;

% Relative growth rate:
RGR=dFFW_FG_V1/FFW_old;
FFW_old = dFFW_FG_V1 + FFW_old;
```

```
Fruit_to_gFW=(1000000/gMOLHEX)/FFW_old;
Maint_Resp_gFW = Rf_maint * Fruit_to_gFW ; % Maint respiration in umol HEX min-1 g-1 FW
Growth_Resp_gFW= Ds*qg*Fruit_to_gFW; % Growth respiration in umol HEX min-1 g-1 FW

% Storage fluxes are in umol equiv. g-1 FW min-1, and their input concentrations as well
% (so correct back from mM L-1 to umol g-1):
V12=max(0,RGR*SUCvac *(1/uMol_to_mM_vac));
V13=max(0,RGR*GLCvac *(1/uMol_to_mM_vac));
V14=max(0,RGR*FRUvac *(1/uMol_to_mM_vac));
V_vac_store=(V12*gMOLSUC/gMOLHEX+V13+V14); % translate V12 to hex equiv.
V21=RGR * y(16);
V222=RGR*y(15);

%% ALL IN UMOL EQUIVALENTS, either SUC or HEX

dSUC=V0_umolSUC-V10+V5+V6-V7; % reworked to SUC equiv, NOT in HEX equiv
% halving the V1 flux not needed, because SUC is half a mole HEX:
dGLCcyt= actflS_umolSUC+V7-V2-V15;
dFRUcyt= actflS_umolSUC+V7-V3-V6-V16;
dGLC6P= V2-V18-V17-V23;
dGLC6P_plast= V23-V25;
dFRU6P= (V3+V17+V24-V4-V8-V9);
dUDPGLC= (V19-V6-V4-V26+V26d);
dS6P= (V4-V5);
dSUCvac= V10-(V11+V12);
dGLCvac= V15+V11-V13;
dFRUvac= V16+(V11-V14);
dG1P= (V18-V19) + V25d;
dFBP= (V8+V9-V24-V20_2);
dCellwall= (V26-(V26d+V222));
dStarch= (V25-(V25d+V21));
dSUGcyt= (dSUC+dFRUcyt+dGLCcyt+dS6P+dUDPGLC+dG1P+dGLC6P+dFRU6P+dFBP);

% fat & protein concentration has a forced, linear time pattern:
dFatProteins = -0.001;
CH2Orest=V20_2-Maint_Resp_gFW - Growth_Resp_gFW; % CH2O remains of glycolysis AFTER
                                                  % respiration costs
dSinkAll= (dSUCvac+dGLCvac+dFRUvac) + dSUGcyt + dGLC6P_plast ...
          + V_vac_store +(dCellwall+dStarch)+(V20_2+V222)+V21;

% output vector dy:
dy = [dSUC;dGLCcyt;dFRUcyt;dGLC6P;dGLC6P_plast;dFRU6P;dUDPGLC;dS6P;dSUCvac; ...
dGLCvac;dFRUvac;dFBP;dG1P;dSinkAll;dCellwall;dStarch; dFFW_FG_V1; dFatProteins; ...
Increase_cellnr2;Increase_cell2;dFFW_FG_pot;Tpf;V0_umolSUC+activS_umol; ...
dSinkAll/2;CH2Orest/2];

end
```

```
%%%%%%%%%%%%%%%%%%%%%%%%%%%%%%%%%%%%%%%%%%%%%%%%%%%%
function par = Parameters1()
%%% Fruit Expansion parameters %%%%%%%%%%%%%%%%%%%
par.acnst   = 0.0163;      % Chen et al. 2021
par.qm20    = 0.00042;   % Maintenance respiration coefficient (g CH2O g-1 DM h-1).
                          % from Bertin and Heuvelink, 1993 (tomato fruit)
par.qg      = 0.3;        % growth respiration coefficient (-). Heuvelink, 1995 (tomato fruit)
par.Km      = 0.08;     % Michaelis constant for active transport of sugar (-).
                        % From Milner et al., 1995 (tomato tonoplast sucrose transport)
par.rwtr    = 0.6;        % Water fraction of respired biomass: correct
par.Q10     = 1.4;        % Effect of T C on qm (-). Bertin and Heuvelink, 1993 (tomato fruit)
par.R       = 8.3144;      % the gas constant (cm3 bar mol–1 K–1)
par.Hf      = 0.99;       % relative humidity in the air-filled space within the fruit
par.Lp      = 0.15;     % Phloem hydraulic conductivity for water to be calibrated;
                        % in g cm-2 MPa-1 h-1. Initial estimate (0.15) from Maggio and Joly, 1995
par.Lx      = 0.22*par.Lp;  % MPa; Xylem hydraulic conductivity for water (g cm-2 MPa-1 h-1)
par.PermS   = 0.000036;  % Sucrose permeability coefficient (g cm–2 h–1).
                          % Ruan & Patrick, 1995 (tomato pericarp phloem membrane)
par.Y       = 0.1;        % 0.1 MPa Liu 2017; Threshold hydrostatic pressure for cell growth (MPa)
par.Y0      = 0;          % Lower threshold hydrostatic pressure for cell growth (MPa).
par.fmax    = 0.1;        % Fishman&Genard 0.1 MPa-1 h-1;  Cell wall extensibility (MPa-1 h-1);
par.paa     = 1.059;       % Factor to calculate fruit osmotic potential due to osmoticum other
                           % than sugars based on fruit osmotic potential due to sugars
par.Mm      = 0.025;       % upper threshold sucrose mass flow (umol g-1 FW min-1)
par.Cp      = 0.08;       % Sucrose concentration of the phloem sap (g g-1)
par.con     = 0.008;       % Vapour permeation fruit skin (modified from De Swaef & Steppe)

end

%%%%%%%%%%%%%%%%%%%%%%%%%%%%%%%%%%%%%%%%%%%%%%%%
function param2 = Parameters2()
%------------------- parameters ----------
% General:
param2.gMOLSUC=342.3;
param2.gMOLHEX=180.2;
% V2: GK
param2.KmGLCcyt_HK_GLC = 0.07;
param2.KmATP_HK_GLC = 0.25;
param2.KmFRUcyt_HK_GLC = 10;
param2.KiGLC6P_HK_GLC = 0.1;
param2.KiFRU6P_HK_GLC = 10;
% V3: FK
param2.KmATPfrka = 0.14;
param2.KmFRUfrka = 0.028;
% V4: SPS
param2.KeqSPS = 10.;
param2.KiS6P_SPS = 0.07;
param2.KmFRU6P_SPS = 0.6;
```

```
param2.KiPiSPS = 3.;
param2.KiUDPGLC_SPS = 1.4;
param2.KmUDPGLC_SPS = 1.8;
param2.rVfVrSPS = 1.9;
param2.KmUDP_SPS = 0.3;
param2.KmS6P_SPS = 1;
param2.KiFRU6P_SPS = 0.4;
param2.Pi = 4.7;
% V5: SPase
param2.KmS6P_SPase = 0.1;
param2.VmSPase = 0.125;
% V6: Susy
param2.KiFRUssc = 3.1;
param2.KmSUCssc = 35.9;
param2.KiUDPssc = 0.0871;
param2.KmUDPssc = 0.00191;
param2.KeqSS = 0.5;
param2.KmUDPGLCssc = 0.234;
param2.KmFRUssc = 6.49;
param2.KiSUCssc = 139.;
param2.rVfVrSusy=0.5;
%V7: Neutral Invertase
param2.KiGLUcytNI = 15;
param2.KmSUC_NI   = 10;
param2.KiFRUcytNI = 15;
%V8: Phosphofructokinase (PFK):
param2.FRU6Phalf= 0.758;
param2.ATPhalf= 0.155;
param2.hpfk= 2.3;
param2.ModATPhalf= 2.;
param2.sigma = 0.9;
% V9: PFP
param2.KeqPfP = 3.3;
param2.KmFRU6Ppfp = 1.;
param2.KmFBPpfp = 0.382;
param2.KmPPipfp = 0.12;
param2.KmPipfp = 0.51;
% V10:
param2.Km10Suc    = 1.2;
%V11, Vacuolar Acid Invertase (AI)
param2.KiGLCvacAI = 26;
param2.KmSUCvacAI   = 10;
param2.KiFRUvacAI = 23.5;
% V16
param2.Km16Glc    =  0.4;
% V17: PGI
param2.Km17G6P=0.31;
param2.Km17F6P=0.174;
```

```
param2.rVfVrPGI=0.565;
% V18: PGM
param2.Km18G6P=0.3;
param2.Km18G1P=0.3;
param2.rVfVrPGM=0.0525;
% V19:UGPase
param2.Km19UTP=0.15;
param2.Km19G1P=0.5;
param2.Km19PPi=0.15;
param2.Km19UDPGLC=0.5;
param2.rVfVrUGPase=0.4;
% V20:Aldolase
param2.KmFBPALD=0.015;
% V23: GPT
param2.Km23G6P=0.6;
param2.Km23Piplast=1.1;
param2.Km23G6Pplast=0.6;
param2.Km23Pi=1.1;
param2.r_Pi=30;
param2.PPi=0.034;
% V24: FBPase
param2.KmFBP=0.1;

end

%%%%%%%%%%%%%%%%%%%%%%%%%%%%%%%%%%%%%%%%%%%%%%
function [svp] = Td2svp(Td)
%
% Td2svp:
% Temperature of dry air - to - saturation vapor pressure
%
% Inputs:
% Td (Temperature of air dry bulb) in degrees Celsius as a vector
%
% Outputs:
% svp (water vapor pressure in saturated air) in Pascals as a vector
%
%%%%%%%%%%%%%%%%%%%%%%%%%%%%%%%%%%%%%%%%%%%%%%
%
% Because:
% svp: Saturation vapor pressure, use Td2svp to get from Temperature of dry air
%
% Given:
% Td: Temperature of dry air - use Td2svp to get - saturation vapor pressure
%
%%%%%%%%%%%%%%%%%%%%%%%%%%%%%%%%%%%%%%%%%%%%%%
%
% filename:     Td2svp.m
```

```
% author:        MartinD@eeng.dcu.ie
% creation:      28 Jan. 1999
% revision:      31 Aug. 2000 (Dave Nartin and Jo Breuer)
% function:      Estimate saturation water vapor pressure
%
%%%%%%%%%%%%%%%%%%%%%%%%%%%%%%%%%%%%%%%%%%%%%%
%
% test method: Code walk thru & comparison with ASHRAE table 2 F6.2 1993
% NB: error in C2 & C9 in F93 (factors C2 & C9 in Handbook are wrong)
% Good values at:
% Hyland, R.W. and A. Wexler, 1983, Formulations for the thermodynamic
% properties, ASHRAE Transactions pag 500-519
% and
% Oliviera, J., T.Singh and S. Lorodocky, 1996,Pshchrometrics; Theory and Practice
%  (ASHRAE-publication), but the sign of C2 and C4 are not good
% dependency:  prefer ASHRAE's Hyland & Wexler 0.7% stated accuracy(-50:+50degC)
%         to Gott-Gratch 1945 used widely by the Meteorology community
%         (endorsed by WMO 1946)
%%%%%%%%%%%%%%%%%%%%%%%%%%%%%%%%%%%%%%%%%%%%%%
%
% reference:
% Psychrometric equations 3 & 4 on P6.7 of ASHRAE fundamentals 1993
%         Checked with Hyland & Wexler & "Psychrometrics - Theory & Practice" P.12
%
% alternatives:
% http://www.natmus.min.dk/cons/tp/atmcalc/atmoclc1.htm
% "empirical equations give good approximation", Padfield
%
% perfect gas law formulation, see, Psychrometrics theory and practice
%
%%%%%%%%%%%%%%%%%%%%%%%%%%%%%%%%%%%%%%%%%%%%%%
% Test Cases
%%%%%%%%%%%%%%%%%%%%%%%%%%%%%%%%%%%%%%%%%%%%%%

% test case for Table 1, Fundamentals, Psychrometric chapter and/or table 7,
% in Psychrometrics theory and practice

%Tdcold = -60:8:-4;
%Tdwarm = 0:8:90;
%Td = [Tdcold Tdwarm];
%Tdc = Td;
%%%%%%%%%%%%%%%%%%%%%%%%%%%%%%%%%%%%%%%%%%%%%

ZerodegC2degK = 273.15;       % 0deg C in deg K, ASHRAE F6.7 (triple point)

svp = Td .* 0;
lnsvp = svp;
warm = find(Td > 0);
```

```
cold = find(Td <= 0);

%        ASHRAE F6.7 eqn.3 T in K

Td = Td + ZerodegC2degK;

c1 = -5.6745359e+03;
c2 =  6.3925247e+00;
c3 = -9.6778430e-03;
c4 =  6.2215701e-07;
c5 =  2.0747825e-09;
c6 = -9.4840240e-13;
c7 =  4.1635019e+00;

t1 = c1 ./ Td(cold);
t2 = c2 ;
t3 = c3 .* Td(cold);
t4 = c4 .* (Td(cold).^2);
t5 = c5 .* (Td(cold).^3);
t6 = c6 .* (Td(cold).^4);
t7 = c7 .* log(Td(cold));

lnsvp(cold) = t1 + t2 + t3 + t4 + t5 + t6 + t7;
svp(cold) = exp(lnsvp(cold));

%ASHRAE F6.7 eqn. 4: T in K and vp in kPa not Pa as noted and confirmed by email by ASHRAE

c8 = -5.8002206e+03;
c9 = 1.3914993e+00;
c10 = -4.8640239e-02;
c11 =  4.1764768e-05;
c12 = -1.4452093e-08;
c13 =  6.5459673e+00;

t8 = c8 ./ Td(warm);
t9 = c9 ;
t10 = c10 .* Td(warm);
t11 = c11 .* (Td(warm).^2);
t12 = c12 .* (Td(warm).^3);
t13 = c13 .* log(Td(warm));

lnsvp(warm) = t8 + t9 + t10 + t11 + t12 + t13;
svp(warm) = exp(lnsvp(warm));

%%%%%%%%%%%%%%%%%%%%%%%%%%%%%%%%%%%%%%%%%%%%%%%
% Test Results
%%%%%%%%%%%%%%%%%%%%%%%%%%%%%%%%%%%%%%%%%%%%%%%
```

```
%format long g;
%resultcold = [Tdc(cold)' Td(cold)' svp(cold)']
%resultwarm = [Tdc(warm)' Td(warm)' svp(warm)']


%resultcold =
%       T in deg C      T in deg K       SVP in Pa
%             -60          213.15       1.08167316646345
%             -52          221.15       3.07018594917586
%             -44          229.15       8.10509034824689
%             -36          237.15       20.0444983678081
%             -28          245.15       46.7298371452917
%             -20          253.15       103.260378580504
%             -12          261.15       217.322502462432
%              -4          269.15       437.475062842583
%               0          273.15       611.153570890768

%resultwarm =
%               8          281.15       1072.84048989454
%              16          289.15       1818.43955751458
%              24          297.15       2985.12706793757
%              32          305.15       4758.53423899428
%              40          313.15       7383.46000898612
%              48          321.15       11175.0878686683
%              56          329.15       16530.4400420306
%              64          337.15       23939.7270257034
%              72          345.15       33997.2585145146
%              80          353.15       47411.6114631381
%              88          361.15       65014.7983487482

%return;
end
```
